# Researcher profiles, proposal submission, and project approval: an exploratory case study at a Brazilian Federal Institute

**DOI:** 10.3389/frma.2026.1888607

**Published:** 2026-10-05

**Authors:** Woska Pires da Costa, Cristhian Chagas Ribeiro, Marcos de Moraes Sousa, Cleiton Mateus Sousa, Priscilla Rayanne e Silva, Kodhandaraman Chinnathambi, Manuel Monfort-Pañego, Matias Noll

**Affiliations:** 1 Research Department, Instituto Federal Goiano - Campus Morrinhos, Morrinhos, Brazil; 2 Faculty of Medicine, Universidade de São Paulo Faculdade de Medicina, São Paulo, Brazil; 3 Research Department, Instituto Federal Goiano - Campus Ceres, Ceres, Brazil; 4 Universidade Estadual de Goiás - Campus Porangatu, Porangatu, Brazil; 5 City Council of Porangatu, Porangatu, Brazil; 6 Universidade Federal de Goiás, Goiânia, Brazil; 7 Instituto Federal Goiano - Campus Urutaí, Urutaí, Brazil; 8 Preparatory Studies Centre, University of Technology and Applied Sciences, Ibra, Oman; 9 Facultat de Magisteri, Universitat de València, Valencia, Spain

**Keywords:** Brazilian Federal Institutes, exploratory case study, grant participation, project approval, proposal submission, research careers, research funding, researcher profiles

## Abstract

**Introduction:**

Decisions to enter funding competitions and subsequent funding outcomes are connected components of a broader process. However, evidence on how researcher-profile characteristics relate to these stages remains limited in public research institutions outside the research-intensive university sector, particularly in emerging-economy research systems.

**Objective:**

This exploratory case study analyzed the associations between researcher-profile characteristics and two respondent-level outcomes among researchers at a Brazilian Federal Institute: proposal submission and, among submitters, self-reported approval of at least one project.

**Methods:**

An online cross-sectional survey was completed by 83 of 327 eligible researchers (response rate 25.4%) in 2023. Data from this self-selected, single-institution sample were analyzed using bivariate contingency-table tests, exact tests for sparse tables, and Cramér's *V* as a descriptive effect-size measure. Findings were treated as hypothesis-generating.

**Results:**

Proposal submission was associated with education level (*p* = 0.020; *V* = 0.285) and teaching in a master's or doctoral degree program (*p* < 0.001; *V* = 0.409). Among respondents who submitted proposals, reporting at least one approved project was associated with age group (*p* = 0.011; *V* = 0.403) and *CNPq* productivity fellowship (*PQ*) status (*p* = 0.047; *V* = 0.284). The approval outcome was measured at the respondent level and did not represent a proposal-level success rate.

**Conclusion:**

By distinguishing submission from approval, this study contributes to research evaluation by indicating that proposal submission and reporting at least one approved project among submitters were associated with different researcher-profile characteristics. These exploratory findings do not establish barriers or causal mechanisms. Multicenter, proposal-level, and longitudinal studies using explicit, context-sensitive definitions of career stage should test whether these patterns vary across institutions, fields, and career trajectories.

## 1 Introduction

Scientific research depends on the allocation of human, material, institutional, and financial resources. These resources shape who can conduct research, which questions are pursued, and how scientific careers develop (Stephan, 2012). However, these resources remain unevenly distributed across countries, institutions, disciplines, and researcher groups (Chankseliani, 2023; Docampo and Cram, 2017; UNESCO, 2021). According to the UNESCO Science Report, about four out of five countries invested < 1% of their gross domestic product (GDP) in research and development (R&D) between 2014 and 2018, whereas the G20 countries and the European Union accounted for approximately 90% of global R&D spending (UNESCO, 2021). These asymmetries are particularly relevant in emerging economies and public research systems, where institutions often combine teaching, research, extension, innovation, and regional development under constrained funding conditions. Funding ecosystems are also heterogeneous. Resources may originate from national research councils and ministries, philanthropic foundations, bilateral and multilateral or development organizations, international programs, universities, and private-sector actors; these sources differ in scope, duration, collaboration requirements, and links to research outputs (Chankseliani, 2023; Thelwall et al., 2023; McManus and Baeta Neves, 2021). International sources can be particularly consequential where domestic research funding is limited, although their importance varies across countries, institutions, fields, and career trajectories.

Research funding is therefore more than a financial input. It operates as a structuring mechanism of scientific activity, influencing productivity, visibility, academic recognition, and career progression (Bloch et al., 2014; Heyard and Hottenrott, 2021; Horta et al., 2016; Rosenbloom et al., 2015; Yan et al., 2018). Competitive grants have been associated with publication output, citation impact, and scientific visibility, although the relationship between funding and impact is not uniform (Heyard and Hottenrott, 2021; Ou et al., 2024). Funding can also amplify differences in reputation, institutional position, and accumulated academic capital, particularly when prior success becomes a criterion for future opportunities (Bol et al., 2018; Franssen and Rijcke, 2019). This dynamic is closely related to cumulative advantage in science. Early success in grant competitions can increase the probability of subsequent funding, even among researchers with similar initial evaluation scores (Bol et al., 2018). Conversely, researchers who do not receive early support may be less likely to participate in future competitions, thereby reinforcing stratification over time (Bol et al., 2018; Franssen and Rijcke, 2019). This is particularly relevant for early-career researchers, because funding requirements that rely heavily on prior grants, consolidated outputs, or established networks may discourage proposal submission before formal peer review begins (Ribeiro et al., 2025).

A growing body of literature shows that funding participation and success are shaped by multiple factors, including age, gender, academic rank, research field, productivity, institutional affiliation, collaboration networks, prior funding experience, and national funding structures (Ebadi and Schiffauerova, 2015; Rusu et al., 2022; Sandström and Van den Besselaar, 2018; Zacharewicz et al., 2019). Collaboration may improve competitiveness by facilitating access to expertise, infrastructure, and scientific visibility, but its effects depend on network configuration, institutional context, and the maturity of collaborative relationships (Ebadi and Schiffauerova, 2015; Andrade et al., 2009; Guan et al., 2016). Academic qualifications and institutional engagement may also influence funding dynamics. Doctoral and postdoctoral training can strengthen research design, scientific writing, methodological autonomy, and proposal preparation (Shibayama, 2019; Belavy et al., 2020; Skinnider et al., 2018). Researchers embedded in stronger research environments may also have better access to mentoring, infrastructure, collaboration, and grant-writing experience (Ryazanova and Jaskiene, 2022; Belavy et al., 2020). Teaching in a master's or doctoral degree program—defined in the Brazilian higher education system as a *stricto sensu* postgraduate program, or simply as “postgraduate program”—may therefore encourage proposal submission; however, such engagement does not necessarily guarantee approval, since funding decisions also depend on evaluation criteria, competition intensity, institutional context, and available resources (Rusu et al., 2022; Recio-Saucedo et al., 2022).

Evaluation systems also shape funding decisions. Peer review remains the dominant mechanism for allocating research grants, but it involves uncertainty, subjective judgment, variability in criteria, and limited predictive capacity (Hug and Aeschbach, 2020; Jerrim and Vries, 2020; Recio-Saucedo et al., 2022; Shepherd et al., 2018). Evaluation criteria often include originality, feasibility, methodological rigor, relevance, applicant track record, team expertise, and alignment with funding priorities (Henriksen and Traynor, 1999; Hug and Aeschbach, 2020; Srivastava et al., 2007). Nevertheless, these dimensions may not be applied consistently across panels, disciplines, or institutional contexts (Jerrim and Vries, 2020; Sattler et al., 2015). For research evaluation, it is therefore important to distinguish access to the evaluation process, through proposal submission, from success after evaluation, through project approval.

Gender disparities have been documented in funding success, evaluation scores, award amounts, and cumulative grant access (Albers et al., 2024; Burns et al., 2019; Head et al., 2013; Schmaling and Gallo, 2023; van der Lee and Ellemers, 2015). In some contexts, gender gaps are more evident when reviewers evaluate applicants than when they evaluate the proposed science (Witteman et al., 2019). Evidence also shows racial and ethnic inequities in access to multiple research project grants among highly funded investigators (Nguyen et al., 2023). These findings do not imply that all local funding systems reproduce the same patterns. Nevertheless, they show that apparently merit-based systems may still be shaped by structural barriers and accumulated advantages.

Brazil is Latin America's largest contributor to scientific publication output, but this leading position coexists with regional concentration and institutional asymmetries (UNESCO, 2021; McManus et al., 2021). Brazilian science has expanded in recent decades, but this progress has been affected by recurrent funding instability and federal budget restrictions since 2015, with consequences for research projects, scholarships, infrastructure, and postgraduate education (Angelo, 2019; McManus et al., 2021). These constraints may reduce opportunities for early-career researchers and weaken institutional capacity, especially outside highly consolidated research centers (McManus et al., 2021; Angelo, 2019; Ribeiro et al., 2025). Within this system, the Federal Network for Professional, Scientific and Technological Education (*RFEPCT*) is a nationwide, multicampus network that integrates professional and technological education across educational levels with research, extension, innovation, and regional development missions (Brazil, 2008; Melo et al., 2023). Compared with research-intensive universities, Federal Institutes combine substantial teaching responsibilities with applied and locally embedded scientific and technological activities. Despite the expanding literature on research funding, limited empirical evidence is available on how researcher profiles relate to both proposal submission and respondent-reported approval among submitters in this distinctive Brazilian institutional context.

Within the Brazilian research system, the productivity fellowship (*PQ*) awarded by the National Council for Scientific and Technological Development (*CNPq*) is one of the most visible markers of scientific recognition. The *PQ* is organized into hierarchical levels and awarded to researchers based on consolidated records of productivity, human resource training, project quality, and scientific contributions (Kamdem et al., 2019; Oliveira et al., 2012). However, *PQ* status should not be interpreted as a simple proxy for future funding success. It is better understood as part of a broader system of recognition that may interact with prior productivity, institutional position, scientific networks, and funding opportunities. Prior research has already distinguished researchers' participation in funding competitions from subsequent funding outcomes and has shown that entry into the applicant pool is part of the funding process (Ayoubi et al., 2021; Rusu et al., 2022). The contribution of the present study is therefore contextual rather than conceptual: it applies related respondent-level outcomes to an institution within *RFEPCT*, a setting that remains underrepresented in research and evaluation studies. Proposal submission and reporting at least one approved project among submitters are treated as connected and logically nested respondent-level outcomes because call targeting, agency choice, proposal framing, submission intensity, and prior feedback may shape who enters a competition and what is later observed among submitters.

Therefore, this exploratory case study aimed to analyze the associations between researcher-profile characteristics and two respondent-level outcomes: (1) reporting the submission of at least one proposal during the preceding 5 years and (2) among submitters, reporting the approval of at least one project during the same period. The study describes patterns within the respondent sample and does not estimate proposal-level success rates, identify causal barriers, or claim representativeness of the population. Nevertheless, it provides context-specific, hypothesis-generating evidence on how researcher profiles relate to participation in funding competitions and respondent-reported approval after submission, potentially informing future research, evaluation practices, and institutional support in public research institutions outside the research-intensive university sector.

## 2 Methods

### 2.1 Study design and participants

A quantitative cross-sectional study (Cummings, 2017) was conducted in 2023 as part of the umbrella study Submission and Approval of Research Projects to Funding Agencies' (*SARFA-Bra* study). Eligible participants were researchers affiliated with *Instituto Federal Goiano (IF Goiano)* who, in 2022, were involved in undergraduate scientific, technological, and innovation research projects or taught in a Brazilian postgraduate program. All 327 eligible researchers at this institution were invited to participate voluntarily, and 83 completed the survey (response rate: 25.4%), resulting in a non-probability, self-selected sample. Most respondents held doctoral degrees; however, no standardized classification of early-, mid-, or late-career stages was adopted. Age group and years of research experience were therefore analyzed as separate observed variables rather than combined into inferred career-stage categories. Given the single-institution, self-selected sample, the findings were interpreted as exploratory patterns among respondents rather than as population estimates.

### 2.2 Context

Created under Law No. 11,892/2008, the *RFEPCT* is a nationwide multicampus public network present in all Brazilian states (Brazil, 2008). It vertically integrates professional and technological education across educational levels with research, extension, innovation, and regional-development missions (IF Goiano, 2023; Melo et al., 2023). The study institution is part of this network and is located in the State of Goiás, in the Midwest region of Brazil. It comprises 12 campuses and an innovation hub distributed across several cities in Goiás, includes centers focused on exponential agriculture and bio-inputs, and offers education from high school through doctoral programs (IF Goiano, 2023). These characteristics make it an informative setting for examining research dynamics within Brazil's public professional and technological education system (IF Goiano, 2023; Melo et al., 2023).

Compared with research-intensive universities, Federal Institutes combine substantial teaching responsibilities and a broad mandate for educational and regional development with applied, locally embedded scientific and technological activities. The technological and regional orientation of the study institution may be related to its infrastructure, disciplinary priorities, available funding calls, and researchers' opportunities to submit proposals. These contextual features are therefore relevant to the interpretation of the findings; however, their effects were not directly measured or tested in the present analysis.

The *PQ* constitutes one of Brazil's main individual research funding mechanisms. These fellowships are intended for researchers with a consolidated record of scientific productivity; they are organized into hierarchical levels based on performance-related criteria, including project quality, researcher training, and scientific contributions to the field (Kamdem et al., 2019). The primary objective of the *PQ* is to foster scientific, technological, and innovative production in the country, while also promoting the selection and development of projects that contribute to advancing knowledge (Kamdem et al., 2019). In the present study, *PQ* status was treated as a respondent-level characteristic and a marker of accumulated scientific recognition, rather than as a measure or proxy of proposal-level funding success.

### 2.3 Ethical approval and informed consent

The study received approval from the Research Ethics Committee of *Instituto Federal Goiano* (Protocol *CAAE* No. 67695523.4.0000.0036, substantiated Opinion No. 6.144.987, on June 27, 2023). All participants signed an Informed Consent Form (ICF) in accordance with Brazilian ethical regulations for research involving human subjects (Brazil, 2012). Participants were informed about the study objectives, methods, and potential risks, and they were assured of their right to withdraw from the study at any time and to decline answering any question.

### 2.4 Data collection instrument

The data were collected through the online “*IF Goiano* Researcher's Profile” questionnaire (Supplementary Data Sheets 1 and 2 – original and English-translated versions, respectively). The full instrument contained one open-ended question and 147 closed-ended questions. For the present analysis, only the items selected through the questionnaire's branching logic were considered, according to each participant's response pathway. Although the questionnaire covered broader dimensions of researchers' work and quality of life, the present analysis focused only on variables directly related to researchers' profiles, proposal submissions, and project approvals. The analyzed parameters were divided into the following blocks:

1. Sociodemographic information (age group, gender, ethnicity, marital status, and children).
2. Professional information (occupation, work unit, employment relationship, workload, other roles at the institution, and work and workload at other institutions).
3. Education and academic qualifications (education level, studies abroad, and teaching in a postgraduate program). The relevant questionnaire item asked whether the respondent taught in any postgraduate program (yes/no); it did not measure program coordination, student supervision, or other forms of participation.
4. Research and teaching activities (time allocated to research, participation in research projects, and *PQ* status).
5. International cooperation (scientific publications, funding, and projects submitted to foreign agencies).

### 2.5 Data collection procedure

All eligible participants were invited to participate in the study via email addresses obtained from the official institutional website. Data collection took place between August 17 and October 6, 2023, via an online questionnaire. Participation was voluntary, and procedures were adopted to preserve confidentiality and anonymity throughout the study. Respondent-level profile data for the 244 nonrespondents were not available in the analytic dataset. Consequently, a respondent–nonrespondent comparison could not be performed, and the magnitude or direction of potential nonresponse bias cannot be determined.

### 2.6 Outcomes

The primary outcomes were respondent-level reports of proposal submission and of at least one approved project among submitters. Proposal submission was coded as a binary indicator of whether the respondent reported having submitted at least one research proposal to a funding call during the preceding 5 years. The second outcome was evaluated only among submitters and indicated whether the respondent reported at least one approved project during the same period. Thus, the outcomes are logically nested rather than independent. The approval indicator is not a proposal-level success rate, does not distinguish respondents who submitted once from those who submitted repeatedly, and does not capture the funding agency, funding amount, evaluation score, resubmission status, or competition intensity.

### 2.7 Data analysis

Absolute and relative frequencies were used to characterize the sample. Associations between researcher-profile variables and the two outcomes (proposal submission and project approval) were examined using Pearson's chi-square test when expected-frequency assumptions were adequate (α = 0.05). When expected cell frequencies were low, two-sided Fisher's exact tests were used for sparse 2 × 2 tables, and Fisher–Freeman–Halton exact tests, with Monte Carlo estimation when needed, were used for larger sparse contingency tables (Agresti, 2019; Hope, 1968; Freeman and Halton, 1951).

For statistically significant associations, Cramér's *V* was calculated from the Pearson χ^2^ statistic as a descriptive measure of effect size for contingency tables. Values range from zero (no association) to one (perfect association). Because all displayed comparisons had min (*r*−1, *c*−1) = 1, conventional Cohen-type benchmarks were used as approximate interpretive references: “very small” (< 0.10), “small” (from 0.10 to < 0.30), “moderate” (from 0.30 to < 0.50), and “large” (≥0.50) (Cramér, 1999; Cohen, 2013). These categories are heuristic; the estimates are descriptive and unadjusted and should be interpreted cautiously given the sample size and sparse-cell structure (Agresti, 2019; Bergsma, 2013).

When contingency tables larger than 2 × 2 showed statistically significant associations, cellwise adjusted standardized residuals were inspected to identify the cells contributing most to the association, using |1.96| as the conventional threshold and interpreting *post hoc* findings cautiously in light of error-rate considerations (MacDonald and Gardner, 2000). Because multiple bivariate comparisons were performed, *p*-values were interpreted as exploratory and hypothesis-generating rather than as evidence of independent predictors. Ultimately, all statistical analyses were performed using IBM™ SPSS™, version 26.0 for Microsoft Windows™ (IBM Corp., Armonk, NY, USA).

## 3 Results

Among respondents, most were male (61.4%), self-identified as white (53.0%), and aged between 34 and 45 years (55.4%). Professionally, most were faculty members/professors (88.0%), held a doctoral degree with or without postdoctoral experience (91.6%), and had a weekly workload exceeding 40 h (53.0%). There was high participation in research projects (96.4%). Nevertheless, only 10.8% held a *PQ*. The largest proportion of respondents (47.0%) allocated 11–20 h per week to teaching activities. Participation in international cooperation was reported by 32.5% of respondents, and only 4.8% had submitted projects to foreign funding agencies. Over the previous 5 years, 74.7% of respondents had submitted at least one research project to funding agencies. The largest subgroup reported submitting 1–3 projects (37.3%), followed by no submissions (25.3%), 4–6 submissions (22.9%), and ≥7 submissions (14.5%). Among those who had submitted projects, 67.7% reported at least one approved project. Specifically, 56.5% reported 1–3 approved projects, 11.2% reported ≥4 approved projects, and 32.3% reported no approved projects (Table 1).

**Table 1:** Sociodemographic, professional, educational, and research characteristics of sample (*n* = 83).

Parameters	*n*	%
Age group		
28–33 years	16	19.3
34–39 years	24	28.9
40–45 years	22	26.5
≥46 years	21	25.3
Gender		
Female	32	38.6
Male	51	61.4
Ethnicity		
White	44	53.0
Black/Mixed	39	47.0
Marital status		
With a partner	61	73.5
Without a partner	22	26.5
Children		
No	31	37.3
Yes	52	62.7
Occupation at *IF Goiano*		
Teacher	73	88.0
Administrative technician	10	12.0
Employment relationship with *IF Goiano*		
External collaborator	3	3.6
Public servant	80	96.4
The weekly workload at *IF Goiano*		
≤ 40 h	39	47.0
>40 h	44	53.0
Other roles at *IF Goiano*		
No	47	56.6
Yes	36	43.4
Work at other institutions		
No	76	91.6
Yes	7	8.4
Workload at other institutions		
≤ 20 h	5	71.4
>20 h	2	28.6
Education level		
Master's degree	7	8.4
Doctorate	55	66.3
Doctorate with postdoctoral	21	25.3
Studies abroad		
No	61	73.5
Yes	22	26.5
Teaching in a postgraduate program		
No	42	50.6
Yes	41	49.4
Time as a researcher		
≤ 10 years	53	63.9
>10 years	30	36.1
Participation in research projects		
No	3	3.6
Yes	80	96.4
*CNPq PQ* status		
No	74	89.2
Yes	9	10.8
Weekly time allocated to teaching		
< 5 h	0	–
5–10 h	15	18.1
11–20 h	39	47.0
≥21 h	29	34.9
Weekly time allocated to research		
< 5 h	8	9.6
5–10 h	29	35.0
11–20 h	25	30.1
≥21 h	21	25.3
Weekly time allocated to extension programs		
< 5 h	46	55.5
5–10 h	28	33.7
11–20 h	9	10.8
≥21 h	0	–
Participation in international cooperation		
No	56	67.5
Yes	27	32.5
Publications in international cooperation		
No	7	25.9
Yes	20	74.1
International cooperation funding		
No	56	67.5
Yes	27	32.5
Proposals submitted to foreign agencies		
No	79	95.2
Yes	4	4.8
Research proposals submitted		
None	21	25.3
1–3	31	37.3
4–6	19	22.9
≥7	12	14.5
Approved projects reported among submitters^*^		
None	20	32.3
1–3	35	56.5
≥4	7	11.2

*n* refers to absolute frequency and % to relative frequency. ^*^Percentages for the reported number of approved projects were calculated among the 62 respondents who reported submitting at least one research proposal during the preceding 5 years. These respondent-level categories are not approval rates per submitted proposal.

Table 2 presents exploratory associations between researcher profiles and the two respondent-level outcomes. Proposal submission was associated with education level (*p* = 0.020; Cramér's *V* = 0.285) and teaching in a postgraduate program (*p* < 0.001; Cramér's *V* = 0.409). Among respondents who submitted proposals, reporting at least one approved project was associated with age group (*p* = 0.011; Cramér's *V* = 0.403) and *PQ* status (*p* = 0.047; Cramér's *V* = 0.284). International cooperation did not meet the prespecified significance threshold (*p* = 0.054). These bivariate findings do not represent adjusted effects or proposal-level approval probabilities.

**Table 2:** Distribution of respondent-reported proposal submission and approval of at least one project among submitters, according to researcher-profile characteristics.

Parameters	At least one proposal submitted		*p*-value	Effect size	At least one approved project among submitters		*p*-value	Effect size
	No	Yes			No	Yes		
Age group								
28–33 years	6 (37.5)	10 (62.5)	0.664^FH^		6 (60.0)#	4 (40.0)	**0.011** ^FH*^	0.403
34–39 years	6 (25.0)	18 (75.0)			1 (5.6)	17 (94.4)#		
40–45 years	5 (22.7)	17 (77.3)			7 (41.2)	10 (58.8)		
≥46 years	4 (19.0)	17 (81.0)			6 (35.3)	11 (64.7)		
Gender								
Female	11 (34.4)	21 (65.6)	0.132^P^		7 (33.3)	14 (66.7)	0.897^P^	
Male	10 (19.6)	41 (80.4)			13 (31.7)	28 (68.3)		
Ethnicity								
White	10 (22.7)	34 (77.3)	0.567^P^		9 (26.5)	25 (73.5)	0.283^P^	
Black/Mixed	11 (28.2)	28 (71.8)			11 (39.3)	17 (60.7)		
Education level								
Master's degree	3 (42.9)	4 (57.1)	**0.020** ^FH*^	0.285	2 (50.0)	2 (50.0)	0.274^FH^	
Doctorate	17 (30.9)	38 (69.1)			14 (36.8)	24 (63.2)		
Doctorate with postdoctoral	1 (4.8)	20 (95.2)#			4 (20.0)	16 (80.0)		
Teaching in a postgraduate program								
No	18 (42.9)#	24 (57.1)	**< 0.001** ^P*^	0.409	10 (41.7)	14 (58.3)	0.208^P^	
Yes	3 (7.3)	38 (92.7)#			10 (26.3)	28 (73.7)		
*CNPq PQ* status								
No	21 (28.4)	53 (71.6)	0.103^F^		20 (37.7)#	33 (62.3)	**0.047** ^F*^	0.284
Yes	–	9 (100.0)			–	9 (100.0)#		
Weekly time allocated to teaching								
< 5 h	–	–	0.317^FH^		–	–	0.237^FH^	
5–10 h	2 (13.3)	13 (86.7)			4 (30.8)	9 (69.2)		
11–20 h	9 (23.1)	30 (76.9)			7 (23.3)	23 (76.7)		
≥21 h	10 (34.5)	19 (65.5)			9 (47.4)	10 (52.6)		
Weekly time allocated to research								
< 5 h	3 (37.5)	5 (62.5)	0.329^FH^		2 (40.0)	3 (60.0)	0.250^FH^	
5–10 h	10 (34.5)	19 (65.5)			9 (47.4)	10 (52.6)		
11–20 h	4 (16.0)	21 (84.0)			6 (28.6)	15 (71.4)		
≥21 h	4 (19.0)	17 (81.0)			3 (17.6)	14 (82.4)		
Participation in international cooperation								
No	17 (30.4)	39 (69.6)	0.127^P^		16 (41.0)	23 (59.0)	0.054^P^	
Yes	4 (14.8)	23 (85.2)			4 (17.4)	19 (82.6)		

*n* refers to absolute frequency and % to relative frequency. For ‘At least one proposal submitted', percentages were calculated using all respondents within each researcher-profile category. For ‘At least one approved project among submitters', percentages were calculated among respondents who reported submitting at least one proposal during the preceding 5 years; the Yes column indicates those who reported at least one approved project and is not a proposal-level approval rate. *p*-values refer to Pearson's χ^2^ tests when expected-frequency assumptions were adequate, two-sided Fisher's exact tests for sparse 2 × 2 tables, and Fisher–Freeman–Halton exact tests with Monte Carlo estimation when needed for larger sparse tables. Cramér's *V*, derived from the Pearson χ^2^ statistic, is reported only for statistically significant associations as an unadjusted, descriptive measure of association. Cramér's *V* ranges from 0 (no association) to 1 (perfect association); conventional benchmarks were used: very small (< 0.10), small (0.10 to < 0.30), moderate (0.30 to < 0.50), and large (≥0.50); these classifications are approximate, and the estimates are descriptive (Cohen, 2013).

The symbol ^#^identifies cells with positive adjusted standardized residuals ≥1.96, indicating observed frequencies higher than expected within statistically significant associations in *post hoc* residual analysis.

^P^refers to Pearson's χ^2^ test; ^F^to Fisher's exact test; and ^FH^to the Fisher–Freeman–Halton exact test.

The *PQ*-holder category comprised 9 respondents in the full sample; therefore, findings regarding *PQ* status should therefore also be interpreted with caution.

Bold values marked with ^*^ indicate statistically significant associations (α = 0.05). The complete distribution of all collected variables is available in Supplementary Table 1.

Figure 1 was prepared directly from the effect sizes and *p*-values reported in Table 2; no additional statistical analysis was performed for the visualization.

**Figure 1:**
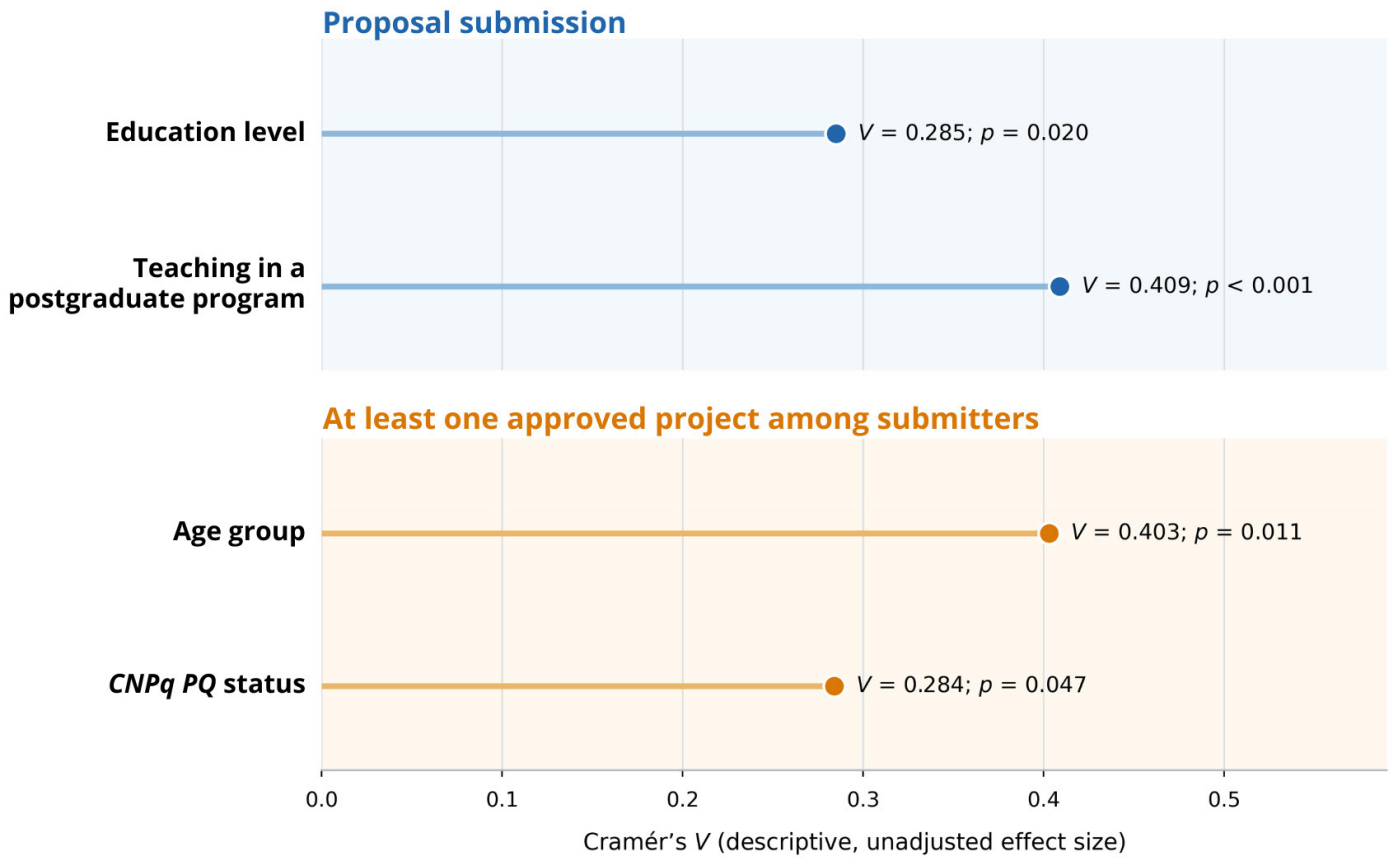
Cramér's *V* for the four principal exploratory associations, separated by respondent-level outcome. The blue upper panel presents associations with proposal submission, whereas the orange lower panel presents associations with reporting at least one approved project among submitters. Approval refers to reporting at least one approved project among respondents who submitted proposals during the preceding 5 years; it is not a proposal-level success rate. Cramér's *V* ranges from 0 (no association) to 1 (perfect association); conventional benchmarks were used: very small (<0.10), small (0.10 to <0.30), moderate (0.30 to <0.50), and large (≥0.50); these classifications are approximate, and the estimates are descriptive (Cohen, 2013) and unadjusted.

## 4 Discussion

### 4.1 Main findings

This exploratory case study identified different respondent characteristics associated with reporting proposal submission and with reporting at least one approved project among submitters. Submission was associated with education level and teaching in a postgraduate program, whereas reporting at least one approval among submitters was associated with age group and *PQ* status (Table 2; Figures 1, 2). These patterns should not be interpreted as direct evidence of barriers, causal effects, independent predictors, or proposal-level success. The outcomes are logically nested and connected within a broader process: researchers decide whether, where, and how often to submit, while observed outcomes among submitters may also reflect proposal framing, agency choice, resubmission, feedback, networks, and prior experience.

**Figure 2:**
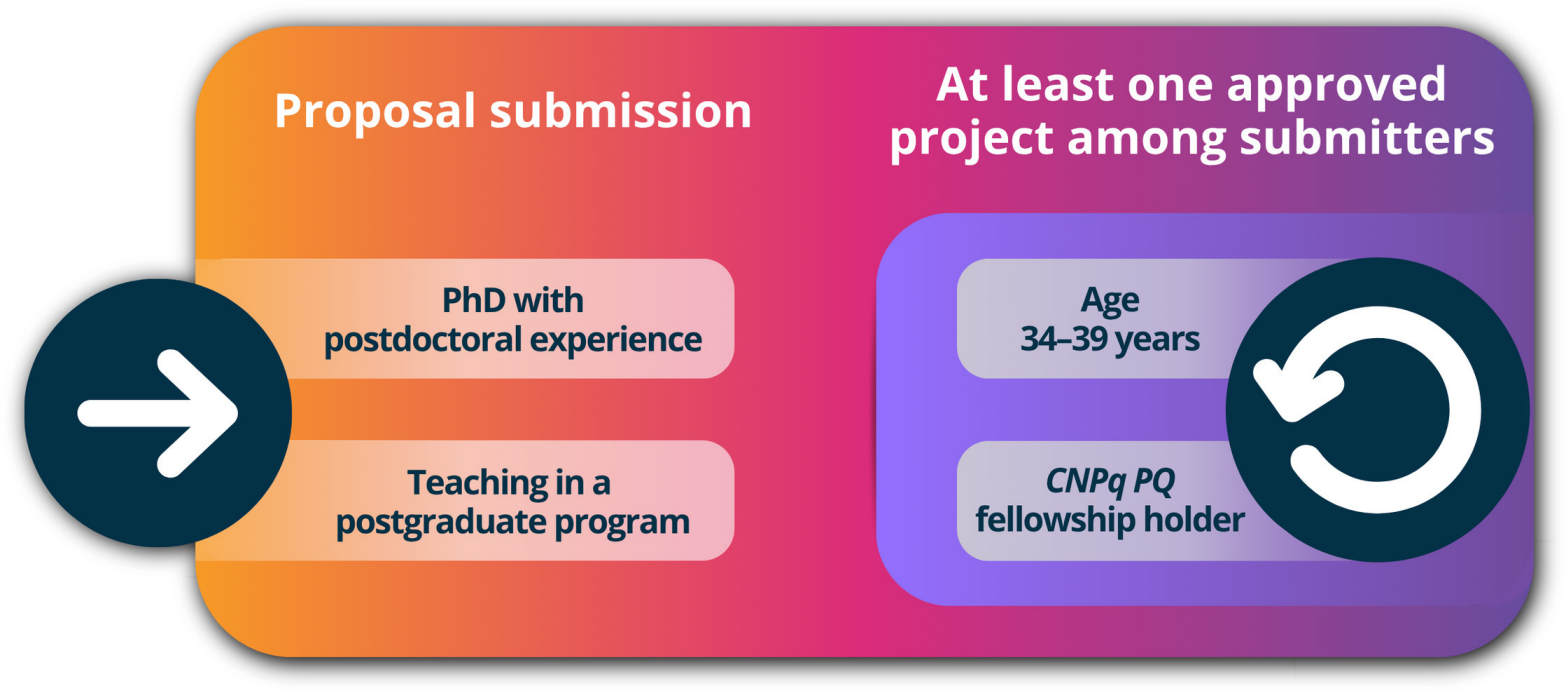
Researcher-related characteristics associated with research proposal submission and reporting at least one approved project among submitters in the context of the study institution in the context of the study institution. *CNPq* stands for the National Council for Scientific and Technological Development, a major federal research funding agency in Brazil; and *PQ* refers to productivity fellowships. Approval refers to reporting at least one approved project among submitters during the preceding five years, not a proposal-level success rate. The highlighted characteristics were analyzed separately and do not define a combined predictive profile.

Among the four principal associations, teaching in a postgraduate program (*V* = 0.409) and age group (*V* = 0.403) had the largest descriptive Cramér's *V* values. Effect-size comparisons remain exploratory because estimates arose from different table structures and denominators, multiple bivariate tests were performed, and several cells were small. The *PQ* result is especially uncertain because only nine respondents held this fellowship in the full sample.

No statistically significant association was detected for several other characteristics, including gender, ethnicity, parenthood, overall weekly workload, time allocated to research, and broad international cooperation indicators. In this small self-selected sample, non-significance should not be interpreted as evidence of equality or absence of meaningful differences; limited power, coarse measurement, nonresponse, and disciplinary composition may obscure patterns.

A processual and relational interpretation is more appropriate than a static barrier model. Academic qualifications, age, and *PQ* status may mark accumulated training, experience, visibility, network position, mentoring, and familiarity with agencies, but none of these mechanisms was directly measured. Likewise, submission and approval are connected through researchers' strategies and learning over time: call targeting, proposal framing, feedback, resubmission, and institutional support can influence both entry into competitions and later outcomes (Ebadi and Schiffauerova, 2015; Franssen and Rijcke, 2019; Wrightson et al., 2025). The observed associations, therefore, generate hypotheses about trajectories and institutional embeddedness rather than identifying deficits among individual researchers.

### 4.2 Proposal submission and academic qualification

Education level was associated with reporting of proposal submission in this sample of respondents. Doctoral and postdoctoral training may provide experience in research design, writing, mentoring, and academic networks, but the present bivariate analysis cannot determine which resources account for the association or whether it persists after accounting for field, career duration, institutional role, and opportunity to submit. The result should therefore be interpreted as a descriptive difference in participation, not as a credential effect or proof of a barrier for less-qualified researchers.

This interpretation is consistent with evidence that research environments and supervisory structures can shape research outcomes. Belavy et al. (2020) found that PhD outcomes were more strongly related to the research environment and experienced supervision than to previous academic performance alone. Similarly, among Canadian MD/PhD graduates, sustained research involvement was positively associated with completion of a research fellowship and research productivity during training (Skinnider et al., 2018). In the present study, academic qualification was associated with submission but not with approval. This pattern is compatible with advanced training being more closely related to entry into funding competitions than to subsequent respondent-reported approval; however, the bivariate design cannot identify the underlying mechanism. Given the small effect size, education level should be interpreted as one contextual marker among field, career duration, institutional role, and opportunity to submit, rather than as an isolated driver of submission.

### 4.3 Teaching in a postgraduate program and proposal submission

Teaching in a postgraduate program was associated with proposal submission. Such teaching may coincide with research-group membership, graduate-student collaboration, publication expectations, mentoring, or awareness of calls; however, these possible pathways were not measured. The questionnaire item captured teaching only and should not be interpreted as evidence of program coordination, supervision, or deeper organizational integration.

Teaching in a postgraduate program was not significantly associated with submitters reporting at least one approved project. This difference does not divide the funding process into independent stages; rather, it indicates that distinct respondent characteristics may be associated with the two logically connected outcomes. Subsequent approval may also reflect unmeasured factors, including proposal quality, alignment with funding-agency priorities, reviewer judgments, funding availability, and prior review or funding-related experience (Recio-Saucedo et al., 2022; Wrightson et al., 2025). The non-significant association does not show that postgraduate teaching is irrelevant to proposal quality or evaluation, because submission intensity, agency choice, proposal characteristics, evaluation scores, and resubmission were not captured.

### 4.4 Age group and any project approval among submitters

The proportion of submitters reporting at least one approved project varied by age group, with the highest observed proportion among respondents aged 34–39 years. The pattern was non-monotonic and should not be translated into an early-, mid-, or late-career effect. Age group may correlate with unmeasured career duration, field, network development, visibility, application intensity, or cohort-specific opportunities; it may also reflect sampling variability in a small institutional sample.

Evidence from other contexts suggests that age and academic rank may be associated with participation and outcomes in funding competitions, although these patterns are embedded in career trajectories and vary across funding systems and disciplines (Rusu et al., 2022). Because definitions of career stage also vary across systems, and this study did not apply a standardized career-stage framework, age group and time were analyzed separately, and respondents were not classified as early- or mid-career researchers. Accordingly, the higher proportion of respondents aged 34–39 years who reported at least one approved project among submitters should be interpreted as an exploratory pattern within this sample rather than as evidence of a universal age- or career-stage effect. Multicenter, longitudinal studies using explicit, context-sensitive career-stage definitions should examine whether this pattern is replicated in other settings.

### 4.5 Productivity fellowships, recognition, and cumulative advantage

*PQ* status was associated with reporting any approved project among submitters. All *PQ* holders in the full sample submitted proposals and reported at least one approved project. This pattern is plausible because *PQ* fellowships are awarded to researchers with a consolidated record of scientific productivity, human resource training, project quality, and recognized contributions to their fields (Kamdem et al., 2019). *PQ* status may therefore reflect accumulated academic capital, visibility, scientific recognition, and prior research trajectories. However, given that only nine respondents held a *PQ* fellowship, the estimate is unstable and cannot establish that fellowship status independently predicts subsequent proposal outcomes.

Evidence on *PQ* holders also cautions against treating fellowship status as homogeneous. Ranking systems based primarily on journal impact factors may not adequately distinguish researchers, and productivity and scientific independence vary across fellowship levels and fields (Oliveira et al., 2012; Kamdem et al., 2019). In this study, the small effect size (Cramér's *V* = 0.284) and the nine-person *PQ* subgroup mean that fellowship status should be interpreted as an unstable marker of accumulated recognition and prior trajectories, not as an independent predictor of later proposal outcomes.

This finding is compatible with cumulative advantage in science and with Merton's formulation of the Matthew effect, in which recognition tends to accumulate around already visible or well-positioned researchers and can shape access to future opportunities (Merton et al., 1968). In a Dutch early-career funding program, Bol et al. (2018) showed that funded researchers accumulated more resources over time than near-winners with similar initial review scores. Funding success may therefore expand later opportunities. However, the present cross-sectional data cannot test this process or establish whether *PQ* status preceded the reported approvals. The observed pattern may reflect cumulative advantage, selection into *PQ* status, greater submission intensity, field composition, or other unmeasured characteristics.

### 4.6 Gender, ethnicity, parenthood, and interpretation of non-significant findings

Gender, ethnicity, and parenthood were not significantly associated with proposal submission or project approval in this sample. This finding should be interpreted carefully. It indicates that no statistically significant association was detected in this dataset, but it should not be presented as evidence that structural inequalities are absent. The sample was limited to one institution and may not have sufficient statistical power to detect subgroup differences.

The broader literature presents mixed and context-dependent evidence. In the U.S. NIH R01 biomedical research funding context, Forscher et al. (2019) found little to no race or gender bias in the initial stage of proposal review. However, other studies have documented gender disparities in success rates, applicant-focused evaluations, award amounts, and access to multiple grants (Burns et al., 2019; Head et al., 2013; Nguyen et al., 2023; van der Lee and Ellemers, 2015; Witteman et al., 2019). For example, Witteman et al. (2019) showed that gender gaps were more evident when the evaluation focused on the applicant rather than the proposed science. Nguyen et al. (2023) also identified gender, racial, and ethnic inequities among investigators holding multiple NIH research project grants.

Thus, the absence of statistically significant associations in the present study should be framed as a local empirical finding, not as confirmation of an equitable funding environment. Future studies with larger samples should examine whether gender, ethnicity, parental status, and their intersections influence participation in and success within funding programs across the *RFEPCT*.

### 4.7 Workload, international cooperation, and research engagement

The weekly teaching load was not significantly associated with reporting at least one approved project among submitters (global exact-test *p* = 0.237). This finding does not establish the absence of an association. Therefore, this result should be considered an exploratory signal rather than evidence that teaching workload independently predicts funding success.

No statistically significant associations were detected for overall weekly workload at *IF Goiano*, time allocated to research, broad research engagement measures, or international cooperation indicators. These measures may be too coarse to capture how time, collaboration, and institutional resources enter funding strategies, and the study was not powered to establish equivalence across categories.

International cooperation remains a relevant topic for future study, but the observed *p* = 0.054 pattern does not meet the prespecified threshold and is not presented as an association. Funding ecosystems include national councils, bilateral and multilateral development agencies, philanthropic foundations, and private-sector actors, which differ across regions and fields (Chankseliani, 2023; Thelwall et al., 2023; McManus and Baeta Neves, 2021). The broad binary indicators used here cannot identify which types of international relationship or funding source matter.

### 4.8 Brazilian public research context

The Brazilian context is central to interpreting these findings. Brazil accounts for the largest share of scientific publication output in Latin America (UNESCO, 2021), while its research system remains marked by funding instability, regional concentration, and institutional asymmetries (McManus et al., 2021). Aggregate national leadership, therefore, coexists with unequal organizational resources, and neither eliminates structural constraints nor supports the generalization of this single-institution result to the broader Brazilian research system. Federal budget restrictions, especially since 2015, have affected research projects, scholarships, postgraduate education, infrastructure, and institutional planning (McManus et al., 2021; Angelo, 2019).

Federal Institutes remain less studied than research-intensive universities. Within the *RFEPCT*, they form a multicampus, vertically integrated system whose professional and technological mission combines professional education, technological training, research, innovation, and regional development (Brazil, 2008; Melo et al., 2023). This organizational model differs from that of research-intensive universities and may shape institutional infrastructure, teaching demands, disciplinary priorities, available funding calls, and researchers' opportunities to submit proposals. However, this study neither measured how these institutional characteristics operate nor compared disciplines. The institution's technological orientation should therefore be regarded as a contextual consideration and a hypothesis for future investigation rather than as an explanation established by the data. Examining funding dynamics in this setting may help clarify patterns of participation and respondent-reported approval and inform institutional strategies to strengthen research capacity, without implying that the observed associations identify causal barriers.

### 4.9 Implications for institutional policy and funding strategies

Taken together, the findings support cautious, low-risk institutional responses rather than remedies for barriers presumed to have been proven. Institutions may evaluate mentoring, internal peer review, grant-writing support, information about diverse calls, feedback on unsuccessful applications, and structured support for resubmission. These strategies are consistent with evidence on institutional grant capacity and learning, but their effectiveness in the study institution should be prospectively evaluated (Shykhnenko and Sbruieva, 2024; Wrightson et al., 2025).

Any pilot intervention should avoid targeting assistance solely on the basis of age, degree, or *PQ* status. A process-oriented program could instead document call selection, proposal development, internal review, submission, external feedback, resubmission, collaborative networks, and agency interactions across career trajectories. Prospective monitoring of participation and proposal-level outcomes by field, campus, career duration, and other equity-relevant characteristics would allow future policy to respond to observed mechanisms rather than to respondent-profile proxies.

### 4.10 Study limitations

This study has limitations. It examined a nonprobability, self-selected sample from a single institution. Profile data for nonrespondents were unavailable, preventing a nonresponse comparison; more research-active individuals may have been more likely to participate. The findings therefore describe respondents and cannot be treated as population estimates for the institution, the *RFEPCT*, or Brazil.

The second outcome of this study was a binary respondent-level indicator of whether a submitter reported at least one approved project during a 5-year recall period, rather than a proposal-level approval rate. Both outcomes were self-reported and may be affected by recall or classification error. The analysis did not account for the number of submissions, funding agency, call, field, amount, evaluation score, resubmission, competition intensity, or the timing of *PQ* attainment relative to reported approvals. The analysis therefore cannot separate evaluation outcomes from submission intensity, accumulated experience, agency choice, strategic behavior, or temporal ordering. These factors may have influenced the observed associations but could not be disentangled in the present analysis.

Finally, the analysis involved multiple bivariate tests, sparse contingency tables, and a small *PQ* subgroup (*n* = 9), without multivariable adjustment. Age was not a validated career-stage measure, disciplinary composition was not modeled. Because the cross-sectional design does not establish temporality and remains susceptible to confounding, the observed associations should be interpreted as hypothesis-generating rather than causal (Abreu, 2026). The value of the study lies in generating context-specific hypotheses and measurement priorities for larger, multicenter, proposal-level, longitudinal, or mixed-methods research.

## 5 Conclusion

In this self-selected single-institution sample, education level and teaching in a postgraduate program were associated with reporting proposal submission, whereas age group and *PQ* status were associated with reporting at least one approved project among submitters. These respondent-level outcomes are distinguishable but connected within a broader funding process and should not be interpreted as proposal-level success rates. The findings do not demonstrate barriers or causal effects; they identify exploratory local patterns that may reflect unmeasured experience, submission intensity, strategies, networks, funding-agency interactions, disciplinary context, and institutional embeddedness.

### 5.1 Recommendations for future research and policy

Future studies should combine multicenter sampling with proposal-level and longitudinal data. Relevant measures include the number and timing of submissions, funding agency and call type, requested amount, evaluation scores, resubmissions, prior feedback, disciplinary field, career duration, institutional role, networks, mentoring, and use of grant support. Where feasible, respondent–nonrespondent comparisons should be planned from administrative covariates. Qualitative interviews or process tracing could then examine how researchers identify calls, frame proposals, learn from review, interact with agencies, and navigate institutional conditions. Any institutional intervention should be evaluated rather than inferred to be effective from the present associations.

## Data Availability

Individual-level data are not publicly available due to privacy and ethical restrictions related to the small institutional sample and the risk of participant identification, in accordance with Brazilian legislation (Resolution *CNS* No. 466, of December 12, 2012). De-identified aggregate data and analysis materials may be made available by the corresponding author upon reasonable request, provided that the request is compatible with the consent conditions, institutional ethics requirements, and applicable data-protection safeguards.
