# Supplementary material for: Researcher profiles, proposal submission, and project approval: an exploratory case study at a Brazilian Federal Institute
Source: Front Res Metr Anal. 2026 Oct 5;11:1888607. doi: 10.3389/frma.2026.1888607 (PMC13677698; doi:10.3389/frma.2026.1888607)
Supplement: Supplementary file 1 [file Data_Sheet_1.pdf]

Supplementary File 1

Survey questionnaire – Brazilian Portuguese original version

Perfil do Pesquisador do IF Goiano

Prezado Pesquisador,

Somos pesquisadores do Instituto Federal Goiano (IF Goiano) e estamos realizando uma pesquisa sobre o FINANCIAMENTO DA PESQUISA CIENTÍFICA E A QUALIDADE DE VIDA DO PESQUISADOR. Os resultados podem fornecer insights úteis para orientar melhores práticas para pesquisadores, agências de fomento e instituições de pesquisa no futuro. Contamos com sua preciosa participação.

Bloco A – DECISÃO DO CONVIDADO

O tempo estimado para responder integralmente o questionário é de cerca de 15 minutos. Desde já, agradecemos imensamente a sua colaboração!

E-mail: ____

Termo de Consentimento Livre e Esclarecido

Este estudo foi aprovado pelo Comitê de Ética em Pesquisa (Protocolo CAAE nº 67695523.4.0000.0036 e Parecer nº 6.144.987). Antes de começar, por favor, acesse o TCLE para decidir sobre sua participação voluntária.

O TCLE está em conformidade com o item IV.5, alínea "a", da Resolução CNS nº 466/2012, cumprindo as exigências do item IV.3. Se você optar por participar, ao selecionar "CONCORDO" e enviar suas respostas, você dará seu consentimento informado e voluntário. Sua participação é essencial para este estudo, mas você tem liberdade para desistir a qualquer momento, sem nenhuma penalidade. Ao selecionar "DISCORDO", não haverá nenhuma penalidade para você.

Após ter lido e compreendido o Termo de Consentimento Livre e Esclarecido (TCLE), você aceita e concorda em participar deste estudo?

◯ **Concordo** ◯ **Discordo**

A indicação do link do CV Lattes permitirá, por nossa parte, extrair sua produção científica, reduzindo o número de perguntas deste questionário e, consequentemente, otimizando o tempo de resposta.

Informe o link para o seu CV Lattes (opcional): ____

Bloco B – DADOS SOCIODEMOGRÁFICOS

b1. Qual é a sua idade? __________ (em anos)

b2. Você se identifica com o gênero:
- ◯ Feminino
- ◯ Masculino
- ◯ Não binário
- ◯ Prefiro não responder
- ◯ Outro: ____

b3. Qual a sua cor de pele/etnia?
◯ Preta ◯ Parda ◯ Branca ◯ Amarela ◯ Indígena

b4. Qual o seu estado civil?
- ◯ Solteiro
- ◯ Casada ou com união estável
- ◯ Divorciado ou separado
- ◯ Viúvo

Bloco C – SOBRE O TRABALHO

c1. Qual o seu local de trabalho? (Indique a unidade principal do IF Goiano em que você está lotado ou com a qual colabora; se você atua em mais de uma unidade, selecione apenas a que considera sua principal.)
- ◯ Reitoria – Goiânia
- ◯ Campus Avançado de Catalão
- ◯ Campus Avançado de Hidrolândia
- ◯ Campus Avançado de Ipamerí
- ◯ Campus Campos Belos
- ◯ Campus Ceres
- ◯ Campus Cristalina
- ◯ Campus Iporá
- ◯ Campus Trindade
- ◯ Campus Morrinhos
- ◯ Campus Rio Verde
- ◯ Campus Urutaí
- ◯ Polo de Inovação

c2. Identificação Profissional:
◯ Docente ◯ Técnico Administrativo

c3. Qual é a sua carga horária de trabalho no IF Goiano?
- ◯ Até 20 horas semanais
- ◯ Mais de 20 horas até 40 horas semanais
- ◯ Mais de 40 horas semanais

c4. Qual é o seu vínculo com o IF Goiano?
- ◯ Servidor efetivo (concursado)
- ◯ Servidor temporário (contrato temporário)
- ◯ Colaborador externo
- ◯ Outro: ____

c5. Qual é o seu tempo de serviço no IF Goiano? __________ (em anos)

c6. Qual é a sua faixa de remuneração mensal bruta?

◯ Até R$ 2.999,99
◯ R$ 3.000,00 a R$ 5.999,99
◯ R$ 6.000,00 a R$ 8.999,99
◯ R$ 9.000,00 a R$ 11.999,99
◯ R$ 12.000,00 a R$ 14.999,99
◯ R$ 15.000,00 a R$ 17.999,99
◯ R$ 18.000,00 ou mais
◯ Prefiro não responder

c7. Pensando na sua remuneração mensal, você diria que está:

◯ Muito satisfeito
◯ Satisfeito
◯ Nem satisfeito nem insatisfeito
◯ Insatisfeito
◯ Muito insatisfeito

c8. Qual é o seu grau de instrução mais alto alcançado até o momento?

◯ Graduação
◯ Pós-graduação lato sensu (especialização)
◯ Mestrado
◯ Doutorado
◯ Pós-doutorado

c9. Você cursou algum nível de ensino fora do Brasil (integral ou parcial)?

◯ Não
◯ Sim, graduação
◯ Sim, pós-graduação lato sensu (especialização)
◯ Sim, mestrado
◯ Sim, doutorado
◯ Sim, pós-doutorado
◯ Outro: ____

c10. Além da sua atividade como docente ou técnico-administrativo, você ocupa algum outro cargo ou função no IF Goiano?

◯ Sim ◯ Não (Pular para a pergunta [c12])

c11. Especifique as outras funções que você desempenha no IF Goiano:

◯ Reitor
◯ Pró-Reitoria ou Diretoria-Geral
◯ Diretoria
◯ Coordenação-Geral, Gerência ou Diretoria-Adjunta
◯ Coordenação
◯ Chefia de Unidade
◯ Chefia de Seção
◯ Chefia de Divisão
◯ Chefia de Setor
◯ Outro: ____

c12. Além do IF Goiano, você atua em outras instituições de ensino superior ou em outra rede de ensino?

◯ Sim ◯ Não (Pular para a pergunta [c14])

c13. Qual é a carga horária semanal de trabalho que você dedica a outras instituições de ensino superior ou à outra rede de ensino, além do IF Goiano?

◯ Até 20 horas semanais
◯ Mais de 20 horas até 40 horas semanais
◯ Mais de 40 horas semanais

c14. Nos últimos 5 anos, quanto tempo, em média, você gastou por semana com atividades de ensino?

◯ Menos de 5 h ◯ 5–10 h ◯ 11–20 h ◯ 21–30 h ◯ Mais de 30 h

c15. Nos últimos 5 anos, quanto tempo, em média, você gastou por semana com atividades de pesquisa?

◯ Menos de 5 h ◯ 5–10 h ◯ 11–20 h ◯ 21–30 h ◯ Mais de 30 h

c16. Nos últimos 5 anos, quanto tempo, em média, você gastou por semana com atividades de extensão?

◯ Menos de 5 h ◯ 5–10 h ◯ 11–20 h ◯ 21–30 h ◯ Mais de 30 h

Pensando na sua rotina de trabalho, por favor, leia as afirmativas abaixo e indique se você concorda muito, concorda, não concorda nem discorda, discorda ou discorda muito com cada uma delas.

c17. Passo a maior parte do meu tempo em tarefas administrativas.

◯ Concordo muito
◯ Concordo
◯ Não concordo nem discordo
◯ Discordo
◯ Discordo muito

c18. Tenho conseguido progredir rapidamente na minha carreira.

◯ Concordo muito
◯ Concordo
◯ Não concordo nem discordo
◯ Discordo
◯ Discordo muito

c19. Tenho tempo suficiente para trabalhar nos meus artigos.

◯ Concordo muito
◯ Concordo
◯ Não concordo nem discordo
◯ Discordo
◯ Discordo muito

c20. Não vale a pena seguir a carreira de pesquisador no Brasil.
- ◯ Concordo muito
- ◯ Concordo
- ◯ Não concordo nem discordo
- ◯ Discordo
- ◯ Discordo muito

c21. Tenho facilidade em obter financiamento para minhas pesquisas.
- ◯ Concordo muito
- ◯ Concordo
- ◯ Não concordo nem discordo
- ◯ Discordo
- ◯ Discordo muito

c22. Minha carga horária de atividades de docência é muito alta.
- ◯ Concordo muito
- ◯ Concordo
- ◯ Não concordo nem discordo
- ◯ Discordo
- ◯ Discordo muito

c23. Minha produção intelectual é bastante prejudicada por outras tarefas.
- ◯ Concordo muito
- ◯ Concordo
- ◯ Não concordo nem discordo
- ◯ Discordo
- ◯ Discordo muito

c24. Me sinto bastante motivado para trabalhar.
- ◯ Concordo muito
- ◯ Concordo
- ◯ Não concordo nem discordo
- ◯ Discordo
- ◯ Discordo muito

c25. Você atua como professor em algum programa de pós-graduação stricto sensu?

◯ Sim ◯ Não

c26. Há quanto tempo você atua como pesquisador? ___________ (em anos)

c27. Você participa de algum projeto ou grupo de pesquisa?

◯ Sim ◯ Não (Pular para a pergunta [c29])

c28. Qual o seu papel nos grupos de pesquisa? (Marque a opção que representa a função mais atuante que você exerce nos grupos de pesquisa dos quais participa.)

- ◯ Líder (responsável por coordenar e planejar os trabalhos de pesquisa do grupo)
- ◯ Pesquisador (servidor graduado ou pós-graduado envolvido com o desenvolvimento de projetos e produção científica, tecnológica e artística do grupo de pesquisa)
- ◯ Técnico (servidor responsável pelo apoio técnico para a realização de projetos de pesquisa e inovação e para a produção científica, tecnológica e artística do grupo)
- ◯ Estudante (discente que participa ativamente da produção científica e tecnológica vinculada às linhas de pesquisa sob a orientação de pesquisadores do grupo de pesquisa)
- ◯ Membro externo (pesquisador, técnico ou estudante com vínculo ativo em outra instituição de ensino que contribua no desenvolvimento de projetos e produção científica, tecnológica e artística do grupo de pesquisa)

c29. No IF Goiano, você se sente incentivado a realizar pesquisas?

- ◯ Sim
- ◯ Parcialmente (algumas vezes)
- ◯ Não
- ◯ Não sei ou prefiro não responder

c30. Nesta instituição, você se sente incentivado a publicar seus resultados de pesquisa?

- ◯ Sim
- ◯ Parcialmente (algumas vezes)
- ◯ Não
- ◯ Não sei ou prefiro não responder

c31. Você tem o hábito de pesquisar para aprimorar seu desempenho na ação docente ou no trabalho como técnico-administrativo?

- ◯ Sim, frequentemente
- ◯ Sim, algumas vezes
- ◯ Raramente
- ◯ Não, nunca

c32. Na sua percepção, o IF Goiano oferece condições adequadas para que os professores e técnicos administrativos desenvolvam competências e atuem na produção do conhecimento?

- ◯ Sim
- ◯ Parcialmente (algumas vezes)
- ◯ Não
- ◯ Não sei ou prefiro não responder

Bloco D – FATORES RELACIONADOS À SUBMISSÃO DE PROJETOS

d1. Nos últimos 5 anos, quantos projetos de pesquisa você submeteu a editais de financiamento?

◯ Nenhum (Pular para a pergunta [f1])
◯ De 1 a 3 ◯ De 10 a 12
◯ De 4 a 6 ◯ De 13 a 15
◯ De 7 a 9 ◯ 16 ou mais

d2. Quais são os principais motivos que influenciam a sua decisão de submeter projetos de pesquisa a editais de financiamento? (Marque todas as opções aplicáveis.)

◯ Possibilidade de obter financiamento para executar o projeto
◯ Ampliar o impacto e a visibilidade da pesquisa
◯ Acesso a recursos e infraestrutura adicionais
◯ Fortalecimento do currículo acadêmico
◯ Estímulo institucional para submissão de projetos
◯ Outro: ____

d3. Quais são as principais dificuldades enfrentadas ao submeter projetos de pesquisa a editais de financiamento? (Marque todas as opções aplicáveis.)

◯ Requisitos e critérios complexos dos editais
◯ Disponibilidade limitada de recursos de financiamento
◯ Concorrência com outros pesquisadores
◯ Dificuldade em encontrar parcerias institucionais
◯ Dificuldade em encontrar parcerias de pesquisadores colaboradores
◯ Tempo e esforço exigidos na elaboração do projeto
◯ Outro: ____

d4. Quando você deixa de submeter a algum edital, mesmo tendo interesse, qual é o principal motivo da não submissão?

◯ Falta de conhecimento sobre os editais de fomento disponíveis
◯ Dificuldade em encontrar parcerias ou colaboradores para o projeto
◯ Restrições de tempo para elaboração e submissão de projetos
◯ Dificuldade em atender aos critérios e requisitos dos editais
◯ Falta de orientação e suporte institucional para submissão de projetos
◯ Outro: ____

Bloco E – FATORES RELACIONADOS À APROVAÇÃO DE PROJETOS

e1. Nos últimos 5 anos, quantos projetos de pesquisa submetidos por você foram aprovados em editais de financiamento?

◯ Nenhum (Pular para a pergunta [g1])
◯ De 1 a 3 ◯ De 7 a 9
◯ De 4 a 6 ◯ 10 ou mais

e2. Após quantas submissões a editais de fomento você conseguiu financiar sua primeira pesquisa?

◯ Na primeira vez ◯ De 4 a 5 ◯ De 8 a 9
◯ De 2 a 3 ◯ De 6 a 7 ◯ 10 ou mais

e3. Quais estratégias você utiliza para aumentar as chances de aprovação dos seus projetos de pesquisa em editais de financiamento? (Marque todas as opções aplicáveis.)

◯ Buscar parcerias institucionais ou colaborativas
◯ Elaborar um projeto claro e bem estruturado
◯ Adaptar o projeto aos critérios e objetivos do edital
◯ Realizar revisões e ajustes com base em feedbacks anteriores
◯ Demonstrar a relevância e impacto potencial do projeto
◯ Outro: ____

e4. Na sua percepção, quais são os principais critérios que influenciam a aprovação de projetos em editais de financiamento? (Marque todas as opções aplicáveis.)

◯ Mérito científico do projeto
◯ Relevância e impacto do projeto para a sociedade
◯ Experiência e qualificação dos pesquisadores envolvidos
◯ Viabilidade técnica e metodológica do projeto
◯ Alinhamento com os objetivos e prioridades do edital
◯ Outro: ____

e5. Na sua opinião, quais são os principais desafios na obtenção de aprovação de projetos em editais de financiamento? (Marque todas as opções aplicáveis.)

◯ Concorrência acirrada com outros projetos
◯ Restrições orçamentárias e disponibilidade limitada de recursos
◯ Complexidade dos requisitos e critérios dos editais
◯ Dificuldade em encontrar parcerias colaborativas
◯ Tempo e esforço exigidos na elaboração da proposta
◯ Outro: ____

e6. Que tipo de apoio institucional você considera necessário para aumentar as chances de aprovação de projetos em editais de financiamento? (Marque todas as opções aplicáveis.)

◯ Orientação na elaboração de propostas
◯ Recursos financeiros para contrapartida ou custeio do projeto
◯ Capacitações específicas sobre elaboração de projetos e editais
◯ Estímulo à formação de parcerias e redes de colaboração
◯ Outro: ____

Bloco F – FATORES RELACIONADOS À NÃO SUBMISSÃO DE PROJETOS

f1. Nos últimos 5 anos, quais instituições foram responsáveis por financiar seus projetos de pesquisa? (Liste cada instituição separada por vírgula.)

____

____

____

f2. Nos últimos 5 anos, qual foi o montante total de financiamento que você obteve para suas pesquisas? (Escreva no seguinte formato: R$ 70.000,00) ___________________________

f3. Qual é o principal motivo pelo qual você ainda não submeteu nenhum projeto de pesquisa a editais de fomento? (Depois, pular para a pergunta [h1])

- ◯ Falta de conhecimento sobre os editais de fomento disponíveis
- ◯ Dificuldade em encontrar parcerias ou colaboradores para o projeto
- ◯ Restrições de tempo para elaboração e submissão de projetos
- ◯ Dificuldade em atender aos critérios e requisitos dos editais
- ◯ Falta de orientação e suporte institucional para submissão de projetos
- ◯ Outro: ____

Bloco G – FATORES RELACIONADOS À NÃO APROVAÇÃO DE PROJETOS

g1. Quais fatores você acredita que contribuíram para a não aprovação dos seus projetos de pesquisa? (Selecione todas as opções que se aplicam.)

- ◯ A proposta do projeto não estava claramente formulada
- ◯ Falta de experiência prévia na área de pesquisa
- ◯ O projeto era considerado de baixa relevância ou impacto pela agência de fomento
- ◯ Falta de recursos ou infraestrutura adequados para executar o projeto
- ◯ Dificuldades na redação da proposta (por exemplo, escrita científica)
- ◯ O orçamento apresentado para o projeto foi considerado inadequado
- ◯ A proposta do projeto não contemplava parcerias nacionais
- ◯ A proposta do projeto não contemplava parcerias internacionais
- ◯ Outro: ____

Bloco H – FATORES RELACIONADOS À SUBMISSÃO E APROVAÇÃO

A partir de sua experiência sobre submissões e aprovações de projetos de pesquisa em agências de fomento, por favor, indique o quanto você concorda com as seguintes afirmativas:

h1. A clareza da proposta de pesquisa é um fator crítico para a aprovação de projetos de pesquisa.

- ◯ Concordo muito
- ◯ Concordo
- ◯ Não concordo nem discordo
- ◯ Discordo
- ◯ Discordo muito

h2. A relevância e originalidade do projeto de pesquisa são essenciais para a sua aprovação.

- ◯ Concordo muito
- ◯ Concordo
- ◯ Não concordo nem discordo
- ◯ Discordo
- ◯ Discordo muito

h3. A expertise do pesquisador ou da equipe de pesquisa é um fator significativo na aprovação de projetos de pesquisa.

◯ Concordo muito
◯ Concordo
◯ Não concordo nem discordo
◯ Discordo
◯ Discordo muito

h4. A disponibilidade de recursos (por exemplo, instalações de laboratório, equipamentos etc.) no local de pesquisa impacta a aprovação do projeto de pesquisa.

◯ Concordo muito
◯ Concordo
◯ Não concordo nem discordo
◯ Discordo
◯ Discordo muito

h5. A aderência do projeto às diretrizes éticas é um fator importante para a aprovação de um projeto de pesquisa.

◯ Concordo muito
◯ Concordo
◯ Não concordo nem discordo
◯ Discordo
◯ Discordo muito

h6. A existência de parcerias ou colaborações com outras instituições aumenta a probabilidade de aprovação de um projeto de pesquisa.

◯ Concordo muito
◯ Concordo
◯ Não concordo nem discordo
◯ Discordo
◯ Discordo muito

h7. A proporção do orçamento do projeto destinada à execução da pesquisa é um fator importante para a aprovação do projeto de pesquisa.

◯ Concordo muito
◯ Concordo
◯ Não concordo nem discordo
◯ Discordo
◯ Discordo muito

h8. A demonstração de impacto significativo da pesquisa proposta (impacto social, econômico, ambiental etc.) influencia a aprovação do projeto de pesquisa.

◯ Concordo muito
◯ Concordo
◯ Não concordo nem discordo
◯ Discordo
◯ Discordo muito

h9. A compatibilidade do projeto de pesquisa com as prioridades estratégicas da agência de fomento é um fator crítico para sua aprovação.

◯ Concordo muito
◯ Concordo
◯ Não concordo nem discordo
◯ Discordo
◯ Discordo muito

h10. O projeto deve conter um plano de divulgação científica e popularização da ciência.

◯ Concordo muito
◯ Concordo
◯ Não concordo nem discordo
◯ Discordo
◯ Discordo muito

Bloco I – FUGA DE TALENTOS INSTITUCIONAIS

i1. Nos últimos cinco anos, você pensou ou tentou trabalhar em outra instituição no Brasil?

◯ Sim ◯ Não (Pular para a pergunta [i7])

Indique o quanto você concorda com as seguintes afirmativas sobre o motivo de você ter pensado ou tentado trabalhar em outra instituição no Brasil:

i2. Remuneração baixa.

◯ Concordo muito
◯ Concordo
◯ Não concordo nem discordo
◯ Discordo
◯ Discordo muito

i3. Dificuldades em obter financiamento para pesquisas.

◯ Concordo muito
◯ Concordo
◯ Não concordo nem discordo
◯ Discordo
◯ Discordo muito

i4. Falta de tempo e espaço para o desenvolvimento de atividades de pesquisa.

◯ Concordo muito
◯ Concordo
◯ Não concordo nem discordo
◯ Discordo
◯ Discordo muito

i5. Para trabalhar com pesquisadores ou grupos de pesquisa consolidados.

- ◯ Concordo muito
- ◯ Concordo
- ◯ Não concordo nem discordo
- ◯ Discordo
- ◯ Discordo muito

i6. Sentimento de desvalorização como pesquisador.

- ◯ Concordo muito
- ◯ Concordo
- ◯ Não concordo nem discordo
- ◯ Discordo
- ◯ Discordo muito

i7. Nos últimos 5 anos, você pensou ou tentou trabalhar em uma instituição no exterior?

◯ Sim ◯ Não

Indique o quanto você concorda com as seguintes afirmativas sobre o motivo de você ter pensado ou tentado trabalhar em uma instituição no exterior:

i8. Remuneração baixa.

- ◯ Concordo muito
- ◯ Concordo
- ◯ Não concordo nem discordo
- ◯ Discordo
- ◯ Discordo muito

i9. Dificuldades em obter financiamento para pesquisas.

- ◯ Concordo muito
- ◯ Concordo
- ◯ Não concordo nem discordo
- ◯ Discordo
- ◯ Discordo muito

i10. Falta de oportunidades para trabalhar.

- ◯ Concordo muito
- ◯ Concordo
- ◯ Não concordo nem discordo
- ◯ Discordo
- ◯ Discordo muito

i11. Para trabalhar com pesquisadores ou grupos de pesquisa consolidados.

- ◯ Concordo muito
- ◯ Concordo
- ◯ Não concordo nem discordo
- ◯ Discordo
- ◯ Discordo muito

i12. Sentimento de desvalorização como pesquisador.
- ◯ Concordo muito
- ◯ Concordo
- ◯ Não concordo nem discordo
- ◯ Discordo
- ◯ Discordo muito

Bloco J – BOLSISTA DE PRODUTIVIDADE DO CNPQ

j1. Quantas vezes você já se candidatou a uma bolsa de produtividade?
◯ Nenhuma ◯ Uma vez ◯ Duas vezes ◯ Três vezes ◯ Mais de três vezes

j2. Você é bolsista de produtividade do CNPq?
◯ Sim ◯ Não (Pular para a pergunta [j5])

j3. Qual o nível da sua bolsa de produtividade?
◯ Sênior ◯ 1A ◯ 1B ◯ 1C ◯ 1D ◯ 2

j4. Após quantos anos da conclusão do seu doutorado você foi contemplado com a bolsa de produtividade?
◯ 1 ano ◯ 2 anos ◯ 3 anos ◯ 4 anos ◯ 5 anos
◯ 6 anos ◯ 7 anos ◯ 8 anos ◯ 9 anos ◯ 10 anos
◯ Mais de 10 anos depois

j5. Se você está mesmo lendo esta pergunta, marque a opção “Azul”.
◯ Amarelo ◯ Azul ◯ Vermelho ◯ Branco ◯ Verde

Bloco K – USO DE REDES SOCIAIS

Segue abaixo uma lista de redes sociais frequentemente usadas em celulares, computadores e tablets. Por favor, indique qual ou quais dessas redes sociais você usa, ou se não usa nenhuma delas.

k1. WhatsApp:
- ◯ Até 6 vezes por semana
- ◯ 1 a 3 vezes por semana
- ◯ 1 vez a cada 15 dias
- ◯ 1 vez por mês ou menos
- ◯ Não uso esta rede social

k2. Facebook:
- ◯ Até 6 vezes por semana
- ◯ 1 a 3 vezes por semana
- ◯ 1 vez a cada 15 dias
- ◯ 1 vez por mês ou menos
- ◯ Não uso esta rede social

k3. Twitter:

- ◯ Até 6 vezes por semana
- ◯ 1 a 3 vezes por semana
- ◯ 1 vez a cada 15 dias
- ◯ 1 vez por mês ou menos
- ◯ Não uso esta rede social

k4. Instagram:

- ◯ Até 6 vezes por semana
- ◯ 1 a 3 vezes por semana
- ◯ 1 vez a cada 15 dias
- ◯ 1 vez por mês ou menos
- ◯ Não uso esta rede social

k5. LinkedIn:

- ◯ Até 6 vezes por semana
- ◯ 1 a 3 vezes por semana
- ◯ 1 vez a cada 15 dias
- ◯ 1 vez por mês ou menos
- ◯ Não uso esta rede social

k6. YouTube:

- ◯ Até 6 vezes por semana
- ◯ 1 a 3 vezes por semana
- ◯ 1 vez a cada 15 dias
- ◯ 1 vez por mês ou menos
- ◯ Não uso esta rede social

k7. ResearchGate:

- ◯ Até 6 vezes por semana
- ◯ 1 a 3 vezes por semana
- ◯ 1 vez a cada 15 dias
- ◯ 1 vez por mês ou menos
- ◯ Não uso esta rede social

k8. Publons:

- ◯ Até 6 vezes por semana
- ◯ 1 a 3 vezes por semana
- ◯ 1 vez a cada 15 dias
- ◯ 1 vez por mês ou menos
- ◯ Não uso esta rede social

k9. Você costuma divulgar suas pesquisas (artigos publicados, working papers, trabalhos apresentados em congressos etc.) em suas redes sociais?

◯ Sim (Pular para a pergunta [k11]) ◯ Não

k10. Por que não divulga suas pesquisas?

◯ Não acho que vale a pena
◯ Não tenho tempo
◯ Prefiro usar redes sociais para lazer
◯ Divulgação de pesquisas em redes sociais
◯ Outro: ____

k11. A instituição em que você trabalha possui um canal no YouTube?

◯ Sim ◯ Não sei ◯ Não

k12. Esse canal divulga pesquisas científicas (artigos publicados, working papers, trabalhos apresentados em congressos etc.)?

◯ Sim ◯ Não sei ◯ Não

k13. A instituição na qual você trabalha incentiva a divulgação dos trabalhos destacados na página ou na publicação específica do Instituto para a comunidade?

◯ Sim ◯ Não sei ◯ Não

Bloco L – COOPERAÇÃO INTERNACIONAL

Segue abaixo uma lista de idiomas. Por favor, indique o seu grau de fluência em cada um desses idiomas.

l1. Português:

◯ Muito fluente
◯ Fluente
◯ Razoavelmente fluente
◯ Pouco fluente
◯ Sem conhecimento do idioma

l2. Inglês:

◯ Muito fluente
◯ Fluente
◯ Razoavelmente fluente
◯ Pouco fluente
◯ Sem conhecimento do idioma

l3. Espanhol:

◯ Muito fluente
◯ Fluente
◯ Razoavelmente fluente
◯ Pouco fluente
◯ Sem conhecimento do idioma

I4. Francês:

- ◯ Muito fluente
- ◯ Fluente
- ◯ Razoavelmente fluente
- ◯ Pouco fluente
- ◯ Sem conhecimento do idioma

I5. Alemão:

- ◯ Muito fluente
- ◯ Fluente
- ◯ Razoavelmente fluente
- ◯ Pouco fluente
- ◯ Sem conhecimento do idioma

I6. Com que frequência você participa de conversas com pesquisadores ou professores colaboradores em línguas estrangeiras?

- ◯ Todos os dias
- ◯ Algumas vezes por semana
- ◯ Algumas vezes por mês
- ◯ Algumas vezes por ano
- ◯ Nunca

I7. Você já participou de cooperação internacional entre sua instituição e instituições de outros países?

◯ Sim ◯ Não (Pular para a pergunta [I12])

I8. De quantas cooperações internacionais você já participou?

◯ 1 ◯ 2 ◯ 3 ◯ 4 ◯ 5 ◯ 6 ou mais

I9. Escreva abaixo os países das instituições com as quais você já participou de cooperação internacional. (Ex.: França, Alemanha e Finlândia)

____

____

____

____

____

I10. Foram publicados artigos a partir dessas cooperações?

◯ Sim ◯ Não

I11. Qual a forma de financiamento dessas cooperações? (Assinale todas as que se aplicarem.)

- ◯ Bolsa de agência nacional
- ◯ Bolsa de agência internacional
- ◯ Editais nacionais de financiamento
- ◯ Fontes internacionais de financiamento
- ◯ Recursos próprios

l12. Nos últimos 5 anos, você submeteu algum projeto a agências de fomento no exterior?
◯ Sim (Pular para a pergunta [m1]) ◯ Não

l13. Por que não submeteu projetos a agências de fomento no exterior nos últimos cinco anos? (Marque todas as alternativas que se aplicam.)
◯ Falta de oportunidades
◯ Falta de apoio para conseguir a documentação necessária
◯ Dificuldade de identificação de chamadas
◯ Falta de conhecimento sobre as agências disponíveis
◯ Dificuldades linguísticas relacionadas ao idioma do país da agência escolhida
◯ Falta de colaboradores ou parceiros no exterior
◯ Outro: ____

Neste momento estamos finalizando as questões referentes aos tópicos institucionais. As próximas perguntas serão sobre questões pessoais que possam impactar a rotina do pesquisador.

A partir de agora, em mais 7 minutos, você finalizará o questionário.

Bloco M – ESTRUTURA FAMILIAR E O IMPACTO DA MATERNIDADE

m1. Você tem filhos?
◯ Sim ◯ Não (Pular para a questão [n1])

m2. Quantos filhos você tem?
◯ 1 ◯ 2 ◯ 3 ◯ 4 ◯ Mais de 4 filhos

m3. Qual a idade do seu filho mais novo?
◯ Menos de 1 ano
◯ 1 ano ◯ 7 anos ◯ 13 anos
◯ 2 anos ◯ 8 anos ◯ 14 anos
◯ 3 anos ◯ 9 anos ◯ 15 anos
◯ 4 anos ◯ 10 anos ◯ 16 anos
◯ 5 anos ◯ 11 anos ◯ 17 anos
◯ 6 anos ◯ 12 anos ◯ 18 anos ou mais

Pontue, em uma escala de 1 (“nenhum impacto”) a 5 (“muito impacto”), o impacto de ter filhos ou filhas em sua carreira acadêmica nas seguintes atividades.

m4. Produção científica:
◯ 1 ◯ 2 ◯ 3 ◯ 4 ◯ 5

m5. Atividades didáticas:
◯ 1 ◯ 2 ◯ 3 ◯ 4 ◯ 5

m6. Credenciamento e recredenciamento em programas de pós-graduação:
◯ 1 ◯ 2 ◯ 3 ◯ 4 ◯ 5

m7. Atividades de extensão:

◯ 1 ◯ 2 ◯ 3 ◯ 4 ◯ 5

m8. Atividades de divulgação científica:

◯ 1 ◯ 2 ◯ 3 ◯ 4 ◯ 5

m9. Participação em eventos internacionais:

◯ 1 ◯ 2 ◯ 3 ◯ 4 ◯ 5

m10. Participação em eventos nacionais:

◯ 1 ◯ 2 ◯ 3 ◯ 4 ◯ 5

m11. Atividades de orientação:

◯ 1 ◯ 2 ◯ 3 ◯ 4 ◯ 5

m12. Formação de redes de colaboração:

◯ 1 ◯ 2 ◯ 3 ◯ 4 ◯ 5

m13. Coordenação de projetos de pesquisa:

◯ 1 ◯ 2 ◯ 3 ◯ 4 ◯ 5

m14. Liderança de grupo de pesquisa:

◯ 1 ◯ 2 ◯ 3 ◯ 4 ◯ 5

Bloco N – QUALIDADE DE VIDA DO PESQUISADOR

Este questionário trata de como você se sente em relação à sua qualidade de vida, à sua saúde e a outras áreas da sua vida. Por favor, responda todas as questões. Se você não tem certeza sobre qual resposta dar a uma questão, por favor, escolha a alternativa que lhe parece mais apropriada. Esta poderá, muitas vezes, ser a sua primeira escolha.

Por favor, tenha em mente seus valores, aspirações, prazeres e preocupações. Estamos perguntando o que você acha de sua vida, tomando como referência as 2 ÚLTIMAS SEMANAS.

n1. Como você avaliaria sua qualidade de vida? (Por favor, leia cada questão, veja o que você acha e assinale a resposta que lhe parece a melhor.)

◯ Muito ruim ◯ Ruim ◯ Nem ruim nem boa ◯ Boa ◯ Muito boa

n2. Quão satisfeito você está com a sua saúde?

◯ Muito insatisfeito
◯ Insatisfeito
◯ Nem satisfeito nem insatisfeito
◯ Satisfeito
◯ Muito satisfeito

As questões a seguir referem-se ao quanto você tem sentido algumas coisas nas últimas 2 semanas.

n3. Em que medida você acha que sua dor física impede você de fazer o que precisa?

◯ Nada ◯ Pouco ◯ Mais ou menos ◯ Bastante ◯ Extremamente

n4. O quanto você precisa de algum tratamento médico para levar sua vida diária?
◯ Nada ◯ Pouco ◯ Mais ou menos ◯ Bastante ◯ Extremamente

n5. O quanto você aproveita a vida?
◯ Nada ◯ Pouco ◯ Mais ou menos ◯ Bastante ◯ Extremamente

n6. Em que medida você acha que a sua vida tem sentido?
◯ Nada ◯ Pouco ◯ Mais ou menos ◯ Bastante ◯ Extremamente

n7. O quanto você consegue se concentrar?
◯ Nada ◯ Pouco ◯ Mais ou menos ◯ Bastante ◯ Extremamente

n8. O quanto você se sente seguro na sua vida diária?
◯ Nada ◯ Pouco ◯ Mais ou menos ◯ Bastante ◯ Extremamente

n9. Quão saudável é o seu ambiente físico (clima, ruído, poluição, atrativos)?
◯ Nada ◯ Pouco ◯ Mais ou menos ◯ Bastante ◯ Extremamente

As questões seguintes perguntam sobre quão plenamente você tem sentido ou é capaz de fazer certas coisas nestas últimas 2 semanas.

n10. Você tem energia suficiente para seu dia a dia?
◯ Nada ◯ Pouco ◯ Médio ◯ Muito ◯ Completamente

n11. Você é capaz de aceitar sua aparência física?
◯ Nada ◯ Pouco ◯ Médio ◯ Muito ◯ Completamente

n12. Você tem dinheiro suficiente para satisfazer suas necessidades?
◯ Nada ◯ Pouco ◯ Médio ◯ Muito ◯ Completamente

n13. Quão disponíveis estão, para você, as informações de que precisa no seu dia a dia?
◯ Nada ◯ Pouco ◯ Médio ◯ Muito ◯ Completamente

n14. Em que medida você tem oportunidades de realizar atividade de lazer?
◯ Nada ◯ Pouco ◯ Médio ◯ Muito ◯ Completamente

As questões a seguir perguntam sobre quão bem ou satisfeito você se sentiu em relação a vários aspectos de sua vida nas últimas 2 semanas.

n15. Quão bem você é capaz de se locomover?
◯ Muito ruim ◯ Ruim ◯ Nem ruim nem bom ◯ Bom ◯ Muito bom

As questões a seguir perguntam sobre quão bem ou satisfeito você se sentiu em relação a vários aspectos de sua vida nas últimas 2 semanas.

n16. Quão satisfeito você está com o seu sono?
- ◯ Muito insatisfeito
- ◯ Insatisfeito
- ◯ Nem satisfeito nem insatisfeito
- ◯ Satisfeito
- ◯ Muito satisfeito

n17. Quão satisfeito você está com sua capacidade de desempenhar as atividades do seu dia a dia?
- ◯ Muito insatisfeito
- ◯ Insatisfeito
- ◯ Nem satisfeito nem insatisfeito
- ◯ Satisfeito
- ◯ Muito satisfeito

n18. Quão satisfeito você está com sua capacidade de trabalho?
- ◯ Muito insatisfeito
- ◯ Insatisfeito
- ◯ Nem satisfeito nem insatisfeito
- ◯ Satisfeito
- ◯ Muito satisfeito

n19. Quão satisfeito você está consigo mesmo?
- ◯ Muito insatisfeito
- ◯ Insatisfeito
- ◯ Nem satisfeito nem insatisfeito
- ◯ Satisfeito
- ◯ Muito satisfeito

n20. Quão satisfeito você está com suas relações pessoais (amigos, parentes, conhecidos, colegas)?
- ◯ Muito insatisfeito
- ◯ Insatisfeito
- ◯ Nem satisfeito nem insatisfeito
- ◯ Satisfeito
- ◯ Muito satisfeito

n21. Quão satisfeito você está com sua vida sexual?
- ◯ Muito insatisfeito
- ◯ Insatisfeito
- ◯ Nem satisfeito nem insatisfeito
- ◯ Satisfeito
- ◯ Muito satisfeito

n22. Quão satisfeito você está com o apoio que recebe de seus amigos?
◯ Muito insatisfeito
◯ Insatisfeito
◯ Nem satisfeito nem insatisfeito
◯ Satisfeito
◯ Muito satisfeito

n23. Quão satisfeito você está com as condições do local onde mora?
◯ Muito insatisfeito
◯ Insatisfeito
◯ Nem satisfeito nem insatisfeito
◯ Satisfeito
◯ Muito satisfeito

n24. Quão satisfeito você está com o seu acesso aos serviços de saúde?
◯ Muito insatisfeito
◯ Insatisfeito
◯ Nem satisfeito nem insatisfeito
◯ Satisfeito
◯ Muito satisfeito

n25. Quão satisfeito você está com o seu meio de transporte?
◯ Muito insatisfeito
◯ Insatisfeito
◯ Nem satisfeito nem insatisfeito
◯ Satisfeito
◯ Muito satisfeito

As questões a seguir referem-se à frequência com que você sentiu ou experimentou certas coisas nas últimas 2 semanas.

n26. Com que frequência você se sente com sentimentos negativos, como mau humor, desespero, ansiedade e depressão?
◯ Nunca ◯ Algumas vezes ◯ Frequentemente ◯ Muito frequente ◯ Sempre

n27. Este questionário fez você refletir sobre sua atuação enquanto pesquisador ou servidor do IF Goiano?
◯ Totalmente ◯ Muito ◯ Parcialmente ◯ Pouco ◯ Não fez refletir

n28. Há algum comentário final ou algum tópico não abordado neste questionário que você gostaria de mencionar?

____

____

____

____

Agradecemos muito sua participação!
Em breve compartilharemos os resultados da nossa pesquisa.
