# Supplementary material for: Researcher profiles, proposal submission, and project approval: an exploratory case study at a Brazilian Federal Institute
Source: Front Res Metr Anal. 2026 Oct 5;11:1888607. doi: 10.3389/frma.2026.1888607 (PMC13677698; doi:10.3389/frma.2026.1888607)
Supplement: Supplementary file 2 [file Data_Sheet_2.pdf]

Supplementary File 2
Survey questionnaire – English translation version

IF Goiano Researcher Profile

Dear Researcher,

We are researchers at the *Instituto Federal Goiano* (*IF Goiano*), and we are conducting a study on SCIENTIFIC RESEARCH FUNDING AND RESEARCHERS' QUALITY OF LIFE. The results may provide useful insights to guide best practices for researchers, funding agencies, and research institutions in the future. We count on your valuable participation.

Block A – INVITEE'S DECISION

The estimated time to complete the entire questionnaire is approximately 15 minutes. Thank you very much in advance for your cooperation!

Email: ____

Informed Consent Form

This study was approved by the Research Ethics Committee (Protocol CAAE No. 67695523.4.0000.0036 and Opinion No. 6.144.987). Before you begin, please access the ICF to decide on your voluntary participation.

The ICF complies with item IV.5, paragraph "a", of Resolution *CNS* No. 466/2012, and fulfills the requirements of item IV.3. If you choose to participate, by selecting "AGREE" and submitting your answers, you will provide your informed and voluntary consent. Your participation is essential to this study, but you are free to withdraw at any time without any penalty. If you select "DISAGREE", there will be no penalty for you.

After reading and understanding the Informed Consent Form (ICF), do you accept and agree to participate in this study?

◯ **Agree** ◯ **Disagree**

Providing the link to your Lattes CV will allow us to extract your scientific production, reducing the number of questions in this questionnaire and, consequently, optimizing your response time.

Please provide the link to your Lattes CV (optional): ________________________________

Block B – SOCIODEMOGRAPHIC DATA

b1. How old are you? ___________ (in years)

b2. What gender do you identify with?

◯ Female
◯ Male
◯ Non-binary
◯ I prefer not to answer
◯ Other: ____

b3. What is your skin color/ethnicity?

◯ Black ◯ Brown/Mixed ◯ White ◯ Yellow/Asian ◯ Indigenous

b4. What is your marital status?

◯ Single
◯ Married or in a stable union
◯ Divorced or separated
◯ Widowed

Block C – WORK-RELATED DATA

c1. Where do you work? (Indicate the main *IF Goiano* unit to which you are assigned or with which you collaborate. If you work in more than one unit, select only the one you consider to be your main unit.)

◯ Rectorate – Goiânia
◯ Advanced Campus of Catalão
◯ Advanced Campus of Hidrolândia
◯ Advanced Campus of Ipamerí
◯ Campus Campos Belos
◯ Campus Ceres
◯ Campus Cristalina
◯ Campus Iporá
◯ Campus Trindade
◯ Campus Morrinhos
◯ Campus Rio Verde
◯ Campus Urutaí
◯ Innovation Hub

c2. Professional Identification

◯ Teacher/Faculty member ◯ Administrative staff member

c3. What is your weekly workload at *IF Goiano*?

◯ Up to 20 hours per week
◯ More than 20 and up to 40 hours per week
◯ More than 40 hours a week

c4. What is your relationship with *IF Goiano*?

◯ Permanent civil servant (public-service appointment)
◯ Temporary employee (temporary contract)
◯ External collaborator
◯ Other: ____

c5. How long have you worked at *IF Goiano*? ___________ (in years)

c6. What is your gross monthly remuneration range?

◯ Up to R$ 2,999.99
◯ R$ 3,000.00 to R$ 5,999.99
◯ R$ 6,000.00 to R$ 8,999.99
◯ R$ 9,000.00 to R$ 11,999.99
◯ R$ 12,000.00 to R$ 14,999.99
◯ R$ 15,000.00 to R$ 17,999.99
◯ R$ 18,000.00 or more
◯ I prefer not to answer

c7. Thinking about your monthly remuneration, would you say that you are:

◯ Very satisfied
◯ Satisfied
◯ Neither satisfied nor dissatisfied
◯ Dissatisfied
◯ Very dissatisfied

c8. What is the highest level of education you have completed to date?

◯ Undergraduate degree
◯ Postgraduate lato sensu (specialization level)
◯ Master's degree
◯ Doctorate
◯ Postdoctoral training

c9. Have you pursued any level of education outside Brazil fully or partially?

◯ No
◯ Yes, an undergraduate degree
◯ Yes, lato sensu postgraduate (specialization level)
◯ Yes, master's degree
◯ Yes, doctorate
◯ Yes, postdoctoral training
◯ Other: ____

c10. In addition to your work as a faculty member or administrative staff member, do you hold any other position or role at *IF Goiano*?

◯ Yes ◯ No (Skip to question [c12])

c11. Specify the other roles you perform at *IF Goiano*:

◯ Rector
◯ Pro-Rectorate or General Directorate
◯ Directorate
◯ General Coordination, Management, or Deputy Directorate
◯ Coordination
◯ Unit Head
◯ Section Head
◯ Division Head
◯ Department Head
◯ Other: ____

c12. Apart from *IF Goiano*, do you work in other higher education institutions or in another education network?

◯ Yes ◯ No (Skip to question [c14])

c13. How many hours per week do you work in other higher education institutions or in another education network besides *IF Goiano*?

◯ Up to 20 hours per week
◯ More than 20 and up to 40 hours per week
◯ More than 40 hours a week

c14. In the last 5 years, how much time, on average, have you spent per week on teaching activities?

◯ Less than 5 h ◯ 5–10 h ◯ 11–20 h ◯ 21–30 h ◯ More than 30 h

c15. In the last 5 years, how much time, on average, have you spent per week on research activities?

◯ Less than 5 h ◯ 5–10 h ◯ 11–20 h ◯ 21–30 h ◯ More than 30 h

c16. In the last 5 years, how much time, on average, have you spent per week on outreach/extension activities?

◯ Less than 5 h ◯ 5–10 h ◯ 11–20 h ◯ 21–30 h ◯ More than 30 h

Thinking about your work routine, please read the statements below and indicate whether you strongly agree, agree, neither agree nor disagree, disagree, or strongly disagree with each one.

c17. I spend most of my time on administrative tasks.

◯ I strongly agree
◯ I agree
◯ I neither agree nor disagree
◯ I disagree
◯ I strongly disagree

c18. I have been able to progress quickly in my career.

◯ I strongly agree
◯ I agree
◯ I neither agree nor disagree
◯ I disagree
◯ I strongly disagree

c19. I have enough time to work on my articles.

◯ I strongly agree
◯ I agree
◯ I neither agree nor disagree
◯ I disagree
◯ I strongly disagree

c20. Pursuing a research career in Brazil is not worthwhile.

◯ I strongly agree
◯ I agree
◯ I neither agree nor disagree
◯ I disagree
◯ I strongly disagree

c21. I find it easy to obtain funding for my research.

◯ I strongly agree
◯ I agree
◯ I neither agree nor disagree
◯ I disagree
◯ I strongly disagree

c22. My teaching workload is too high.

◯ I strongly agree
◯ I agree
◯ I neither agree nor disagree
◯ I disagree
◯ I strongly disagree

c23. My intellectual output is greatly hampered by other tasks.

◯ I strongly agree
◯ I agree
◯ I neither agree nor disagree
◯ I disagree
◯ I strongly disagree

c24. I feel very motivated to work.

◯ I strongly agree
◯ I agree
◯ I neither agree nor disagree
◯ I disagree
◯ I strongly disagree

c25. Do you teach in any stricto sensu postgraduate program?

◯ Yes ◯ No

c26. How long have you worked as a researcher? __________ (in years)

c27. Do you participate in any research projects or research groups?

◯ Yes ◯ No (Skip to question [c29])

c28. What role do you play in research groups? (Select the option that represents the most active role you play in the research groups in which you participate.)

- ◯ Leader (responsible for coordinating and planning the group's research work)
- ◯ Researcher (graduate or postgraduate staff member involved in the development of projects and in the scientific, technological, and artistic production of the research group)
- ◯ Technician (staff member responsible for providing technical support for research and innovation projects and for the group's scientific, technological, and artistic production)
- ◯ Student (student who actively participates in scientific and technological production linked to the lines of research under the guidance of researchers from the research group)
- ◯ External member (researcher, technician, or student with an active relationship with another educational institution who contributes to the development of projects and to the scientific, technological, and artistic production of the research group)

c29. At *IF Goiano*, do you feel encouraged to conduct research?

- ◯ Yes
- ◯ Partially (sometimes)
- ◯ No
- ◯ I do not know, or I prefer not to answer

c30. At this institution, do you feel encouraged to publish your research results?

- ◯ Yes
- ◯ Partially (sometimes)
- ◯ No
- ◯ I do not know, or I prefer not to answer

c31. Are you in the habit of conducting research to improve your teaching performance or your work as an administrative staff member?

- ◯ Yes, often
- ◯ Yes, sometimes
- ◯ Rarely
- ◯ No, never

c32. In your view, does *IF Goiano* provide adequate conditions for faculty and administrative staff to develop competencies and contribute to knowledge production?

- ◯ Yes
- ◯ Partially (sometimes)
- ◯ No
- ◯ I do not know, or I prefer not to answer

Block D – FACTORS RELATED TO PROJECT SUBMISSION

d1. In the last 5 years, how many research projects have you submitted to funding calls?

◯ None (Skip to question [f1])

◯ From 1 to 3	◯ From 10 to 12
◯ From 4 to 6	◯ From 13 to 15
◯ From 7 to 9	◯ 16 or more

d2. What are the main reasons that influence your decision to submit research projects to funding calls? (Select all that apply.)

◯ Possibility of obtaining funding to carry out the project
◯ Increasing the impact and visibility of the research
◯ Access to additional resources and infrastructure
◯ Strengthening one's academic CV
◯ Institutional encouragement to submit projects
◯ Other: ____

d3. What are the main difficulties faced when submitting research projects to funding calls? (Select all that apply.)

◯ Complex requirements and criteria in the calls
◯ Limited availability of funding resources
◯ Competition with other researchers
◯ Difficulty finding institutional partnerships
◯ Difficulty finding collaborating researchers
◯ Time and effort required to prepare the project
◯ Other: ____

d4. When you do not submit to a funding call, even though you are interested, what is the main reason for not submitting?

◯ Lack of knowledge about available funding calls
◯ Difficulty finding partnerships or collaborators for the project
◯ Time constraints for preparing and submitting projects
◯ Difficulty meeting the criteria and requirements of funding calls
◯ Lack of guidance and institutional support for project submission
◯ Other: ____

Block E – FACTORS RELATED TO PROJECT APPROVAL

e1. In the last 5 years, how many research projects submitted by you were approved in funding calls?

◯ None (Skip to question [g1])

◯ From 1 to 3	◯ From 7 to 9
◯ From 4 to 6	◯ 10 or more

e2. After how many submissions to funding calls did you obtain funding for your first research project?

◯ The first time	◯ From 4 to 5	◯ From 8 to 9
◯ From 2 to 3	◯ From 6 to 7	◯ 10 or more

e3. What strategies do you use to increase the chances of your research projects being approved in funding calls? (Select all that apply.)

- ◯ Seek institutional or collaborative partnerships
- ◯ Prepare a clear and well-structured project
- ◯ Adapt the project to the criteria and objectives of the funding call
- ◯ Make revisions and adjustments based on previous feedback
- ◯ Demonstrate the relevance and potential impact of the project
- ◯ Other: ____

e4. In your perception, what are the main criteria that influence project approval in funding calls? (Select all that apply.)

- ◯ Scientific merit of the project
- ◯ Relevance and impact of the project for society
- ◯ Experience and qualifications of the researchers involved
- ◯ Technical and methodological feasibility of the project
- ◯ Alignment with the objectives and priorities of the funding call
- ◯ Other: ____

e5. In your opinion, what are the main challenges in obtaining project approval in funding calls? (Select all that apply.)

- ◯ Intense competition with other projects
- ◯ Budget constraints and limited availability of resources
- ◯ Complexity of the requirements and criteria of the funding calls
- ◯ Difficulty finding collaborative partnerships
- ◯ Time and effort required to prepare the proposal
- ◯ Other: ____

e6. What kind of institutional support do you consider necessary to increase the chances of project approval in funding calls? (Select all that apply.)

- ◯ Guidance on proposal preparation
- ◯ Financial resources for counterpart funding or project costs
- ◯ Specific training on project preparation and funding calls
- ◯ Encouragement of partnerships and collaborative networks
- ◯ Other: ____

Block F – FACTORS RELATED TO PROJECT NON-SUBMISSION

f1. In the last 5 years, which institutions were responsible for funding your research projects? (List each institution separated by a comma.)

____

____

____

f2. In the last 5 years, what was the total amount of funding you obtained for your research? (Write in the following format: R$ 70,000.00) __________________________

f3. What is the main reason why you have not yet submitted any research project to a funding call? (Then, skip to question [h1])

◯ Lack of knowledge about available funding calls
◯ Difficulty finding partnerships or collaborators for the project
◯ Time constraints for preparing and submitting projects
◯ Difficulty meeting the criteria and requirements of funding calls
◯ Lack of guidance and institutional support for project submission
◯ Other: ____

Block G – FACTORS RELATED TO PROJECT NON-APPROVAL

g1. What factors do you believe contributed to the non-approval of your research projects? (Select all that apply.)

◯ The project proposal was not clearly formulated
◯ Lack of previous experience in the research area
◯ The project was considered to have low relevance or impact by the funding agency
◯ Lack of adequate resources or infrastructure to carry out the project
◯ Difficulties in writing the proposal (e.g., scientific writing)
◯ The budget presented for the project was considered inadequate
◯ The project proposal did not include national partnerships
◯ The project proposal did not include international partnerships
◯ Other: ____

Block H – FACTORS RELATED TO SUBMISSION AND APPROVAL

Based on your experience with the submission and approval of research projects by funding agencies, please indicate how much you agree with the following statements:

h1. The clarity of the research proposal is a critical factor in the approval of research projects.

◯ I strongly agree
◯ I agree
◯ I neither agree nor disagree
◯ I disagree
◯ I strongly disagree

h2. The relevance and originality of the research project are essential for its approval.

◯ I strongly agree
◯ I agree
◯ I neither agree nor disagree
◯ I disagree
◯ I strongly disagree

h3. The expertise of the researcher or research team is a significant factor in the approval of research projects.

◯ I strongly agree
◯ I agree
◯ I neither agree nor disagree
◯ I disagree
◯ I strongly disagree

h4. The availability of resources (e.g., laboratory facilities, equipment, etc.) at the research site affects the approval of the research project.

◯ I strongly agree
◯ I agree
◯ I neither agree nor disagree
◯ I disagree
◯ I strongly disagree

h5. The project's adherence to ethical guidelines is an important factor in the approval of a research project.

◯ I strongly agree
◯ I agree
◯ I neither agree nor disagree
◯ I disagree
◯ I strongly disagree

h6. The existence of partnerships or collaborations with other institutions increases the likelihood of a research project being approved.

◯ I strongly agree
◯ I agree
◯ I neither agree nor disagree
◯ I disagree
◯ I strongly disagree

h7. The proportion of the project budget allocated to conducting the research is an important factor in the approval of the project.

◯ I strongly agree
◯ I agree
◯ I neither agree nor disagree
◯ I disagree
◯ I strongly disagree

h8. Demonstrating the significant impact of the proposed research (social, economic, environmental impact, etc.) influences the approval of the research project.

◯ I strongly agree
◯ I agree
◯ I neither agree nor disagree
◯ I disagree
◯ I strongly disagree

h9. The compatibility of the research project with the funding agency's strategic priorities is a critical factor in its approval.

- ◯ I strongly agree
- ◯ I agree
- ◯ I neither agree nor disagree
- ◯ I disagree
- ◯ I strongly disagree

h10. The project should include a plan for science communication and science popularization.

- ◯ I strongly agree
- ◯ I agree
- ◯ I neither agree nor disagree
- ◯ I disagree
- ◯ I strongly disagree

Block I – INSTITUTIONAL TALENT DRAIN

i1. In the last five years, have you considered or tried to work at another institution in Brazil?

◯ Yes ◯ No (Skip to question [i7])

Please indicate how much you agree with the following statements about why you have considered or tried to work at another institution in Brazil:

i2. Low remuneration.

- ◯ I strongly agree
- ◯ I agree
- ◯ I neither agree nor disagree
- ◯ I disagree
- ◯ I strongly disagree

i3. Difficulties obtaining research funding.

- ◯ I strongly agree
- ◯ I agree
- ◯ I neither agree nor disagree
- ◯ I disagree
- ◯ I strongly disagree

i4. Lack of time and space to develop research activities.

- ◯ I strongly agree
- ◯ I agree
- ◯ I neither agree nor disagree
- ◯ I disagree
- ◯ I strongly disagree

i5. To work with established researchers or research groups.

- ◯ I strongly agree
- ◯ I agree
- ◯ I neither agree nor disagree
- ◯ I disagree
- ◯ I strongly disagree

i6. Feeling undervalued as a researcher.

- ◯ I strongly agree
- ◯ I agree
- ◯ I neither agree nor disagree
- ◯ I disagree
- ◯ I strongly disagree

i7. In the last 5 years, have you considered or tried to work at an institution abroad?

◯ Yes ◯ No

Please indicate how much you agree with the following statements about why you have considered or tried to work at an institution abroad:

i8. Low remuneration.

- ◯ I strongly agree
- ◯ I agree
- ◯ I neither agree nor disagree
- ◯ I disagree
- ◯ I strongly disagree

i9. Difficulties obtaining research funding.

- ◯ I strongly agree
- ◯ I agree
- ◯ I neither agree nor disagree
- ◯ I disagree
- ◯ I strongly disagree

i10. Lack of work opportunities.

- ◯ I strongly agree
- ◯ I agree
- ◯ I neither agree nor disagree
- ◯ I disagree
- ◯ I strongly disagree

i11. To work with established researchers or research groups.

- ◯ I strongly agree
- ◯ I agree
- ◯ I neither agree nor disagree
- ◯ I disagree
- ◯ I strongly disagree

i12. Feeling undervalued as a researcher.
- ◯ I strongly agree
- ◯ I agree
- ◯ I neither agree nor disagree
- ◯ I disagree
- ◯ I strongly disagree

Block J – CNPQ PRODUCTIVITY FELLOWSHIP

j1. How many times have you applied for a productivity fellowship?
◯ Never ◯ Once ◯ Twice ◯ Three times ◯ More than three times

j2. Are you a CNPq productivity fellowship holder?
◯ Yes ◯ No (Skip to question [j5])

j3. What is the level of your productivity fellowship?
◯ Senior ◯ 1A ◯ 1B ◯ 1C ◯ 1D ◯ 2

j4. How many years after completing your doctorate were you awarded the productivity fellowship?
◯ 1 year ◯ 2 years ◯ 3 years ◯ 4 years ◯ 5 years
◯ 6 years ◯ 7 years ◯ 8 years ◯ 9 years ◯ 10 years
◯ More than 10 years later

j5. If you are actually reading this question, select the "Blue" option.
◯ Yellow ◯ Blue ◯ Red ◯ White ◯ Green

Block K – USE OF SOCIAL MEDIA

Below is a list of social media platforms frequently used on cell phones, computers, and tablets. Please indicate which one or ones you use, or whether you do not use any of these social media platforms.

k1. WhatsApp:
- ◯ Up to 6 times a week
- ◯ 1 to 3 times a week
- ◯ Once every 15 days
- ◯ Once per month or less
- ◯ I do not use this social media platform

k2. Facebook:
- ◯ Up to 6 times a week
- ◯ 1 to 3 times a week
- ◯ Once every 15 days
- ◯ Once per month or less
- ◯ I do not use this social media platform

k3. Twitter:

- ◯ Up to 6 times a week
- ◯ 1 to 3 times a week
- ◯ Once every 15 days
- ◯ Once per month or less
- ◯ I do not use this social media platform

k4. Instagram:

- ◯ Up to 6 times a week
- ◯ 1 to 3 times a week
- ◯ Once every 15 days
- ◯ Once per month or less
- ◯ I do not use this social media platform

k5. LinkedIn:

- ◯ Up to 6 times a week
- ◯ 1 to 3 times a week
- ◯ Once every 15 days
- ◯ Once per month or less
- ◯ I do not use this social media platform

k6. YouTube:

- ◯ Up to 6 times a week
- ◯ 1 to 3 times a week
- ◯ Once every 15 days
- ◯ Once per month or less
- ◯ I do not use this social media platform

k7. ResearchGate:

- ◯ Up to 6 times a week
- ◯ 1 to 3 times a week
- ◯ Once every 15 days
- ◯ Once per month or less
- ◯ I do not use this social media platform

k8. Publons:

- ◯ Up to 6 times a week
- ◯ 1 to 3 times a week
- ◯ Once every 15 days
- ◯ Once per month or less
- ◯ I do not use this social media platform

k9. Do you usually share or disseminate your research (published articles, working papers, papers presented at conferences, etc.) on your social media platforms?

◯ Yes (Skip to question [k11]) ◯ No

k10. Why do you not share or disseminate your research?

- ◯ I do not consider it worthwhile
- ◯ I do not have enough time
- ◯ I prefer to use social media for leisure purposes
- ◯ Lack of familiarity with research dissemination on social media
- ◯ Other: ____

k11. Does the institution where you work have a YouTube channel?

◯ Yes ◯ I do not know ◯ No

k12. Does this channel disseminate scientific research (published articles, working papers, papers presented at conferences, etc.)?

◯ Yes ◯ I do not know ◯ No

k13. Does the institution where you work encourage the dissemination, to the community, of work highlighted on the Institute's website or in a specific Institute publication?

◯ Yes ◯ I do not know ◯ No

Block L – INTERNATIONAL COOPERATION

Below is a list of languages. Please indicate your level of fluency in each language.

l1. Portuguese:

- ◯ Very fluent
- ◯ Fluent
- ◯ Reasonably fluent
- ◯ Slightly fluent
- ◯ No knowledge of the language

l2. English:

- ◯ Very fluent
- ◯ Fluent
- ◯ Reasonably fluent
- ◯ Slightly fluent
- ◯ No knowledge of the language

l3. Spanish:

- ◯ Very fluent
- ◯ Fluent
- ◯ Reasonably fluent
- ◯ Slightly fluent
- ◯ No knowledge of the language

l4. French:

◯ Very fluent
◯ Fluent
◯ Reasonably fluent
◯ Slightly fluent
◯ No knowledge of the language

l5. German:

◯ Very fluent
◯ Fluent
◯ Reasonably fluent
◯ Slightly fluent
◯ No knowledge of the language

l6. How often do you participate in conversations with researchers or collaborating faculty members in foreign languages?

◯ Every day
◯ A few times a week
◯ A few times a month
◯ A few times a year
◯ Never

l7. Have you participated in international cooperation (cooperation between your institution and institutions in other countries)?

◯ Yes ◯ No (Skip to question [l12])

l8. How many international cooperation projects have you participated in?

◯ 1 ◯ 2 ◯ 3 ◯ 4 ◯ 5 ◯ 6 or more

l9. Write below the countries of the institutions with which you have participated in international cooperation (e.g., France, Germany, and Finland).

____

____

____

____

____

l10. Have articles been published as a result of these collaborations?

◯ Yes ◯ No

l11. How were these collaborations funded? (Select all that apply.)

◯ Fellowship from a national agency
◯ Fellowship from an international agency
◯ National funding calls
◯ International funding sources
◯ Own resources

l12. In the last 5 years, have you submitted any projects to foreign funding agencies?
◯ Yes (Skip to question [m1]) ◯ No

l13. Why have you not submitted projects to foreign funding agencies in the last five years? (Select all that apply.)
◯ Lack of opportunities
◯ Lack of support to obtain the necessary documentation
◯ Difficulty identifying calls for proposals
◯ Lack of knowledge about available agencies
◯ Language difficulties related to the language of the country of the selected agency
◯ Lack of collaborators or partners abroad
◯ Other: ____

We are now finalizing the questions on institutional topics.
The next questions will address personal matters that may affect the researcher's routine.

From this point, the questionnaire should take about 7 more minutes to complete.

Block M – FAMILY STRUCTURE AND THE IMPACT OF PARENTHOOD

m1. Do you have children?
◯ Yes ◯ No (Skip to question [n1])

m2. How many children do you have?
◯ 1 ◯ 2 ◯ 3 ◯ 4 ◯ More than 4 children

m3. How old is your youngest child?
◯ Less than 1 year

◯ 1 year	◯ 7 years	◯ 13 years
◯ 2 years	◯ 8 years	◯ 14 years
◯ 3 years	◯ 9 years	◯ 15 years
◯ 4 years	◯ 10 years	◯ 16 years
◯ 5 years	◯ 11 years	◯ 17 years
◯ 6 years	◯ 12 years	◯ 18 years or older

Please rate, on a scale from 1 ("no impact") to 5 ("very high impact"), the impact of having children on your academic career in the following activities.

m4. Scientific production:
◯ 1 ◯ 2 ◯ 3 ◯ 4 ◯ 5

m5. Teaching activities:
◯ 1 ◯ 2 ◯ 3 ◯ 4 ◯ 5

m6. Accreditation and re-accreditation in postgraduate programs:
◯ 1 ◯ 2 ◯ 3 ◯ 4 ◯ 5

m7. Outreach/extension activities:
◯ 1 ◯ 2 ◯ 3 ◯ 4 ◯ 5

m8. Science communication activities:
◯ 1 ◯ 2 ◯ 3 ◯ 4 ◯ 5

m9. Participation in international events:
◯ 1 ◯ 2 ◯ 3 ◯ 4 ◯ 5

m10. Participation in national events:
◯ 1 ◯ 2 ◯ 3 ◯ 4 ◯ 5

m11. Advising and supervision activities:
◯ 1 ◯ 2 ◯ 3 ◯ 4 ◯ 5

m12. Building collaborative networks:
◯ 1 ◯ 2 ◯ 3 ◯ 4 ◯ 5

m13. Coordination of research projects:
◯ 1 ◯ 2 ◯ 3 ◯ 4 ◯ 5

m14. Research group leadership:
◯ 1 ◯ 2 ◯ 3 ◯ 4 ◯ 5

Block N – RESEARCHER QUALITY OF LIFE

This questionnaire is about how you feel about your quality of life, health, and other areas of your life. Please answer all questions. If you are unsure which answer to give to a question, please choose the option that seems most appropriate to you. This can often be your first choice.

Please keep in mind your values, aspirations, pleasures, and concerns. We are asking what you think about your life, taking the LAST 2 WEEKS as the reference period.

n1. How would you rate your quality of life? (Please read each question, consider what you think, and select the answer that seems best to you.)
◯ Very bad ◯ Bad ◯ Neither bad nor good ◯ Good ◯ Very good

n2. How satisfied are you with your health?
◯ Very dissatisfied
◯ Dissatisfied
◯ Neither satisfied nor dissatisfied
◯ Satisfied
◯ Very satisfied

The following questions refer to how much you have experienced certain things over the last 2 weeks.

n3. To what extent do you think your physical pain prevents you from doing what you need to do?
◯ Not at all ◯ A little ◯ Moderately ◯ Very much ◯ Extremely

n4. How much medical treatment do you need to function in your daily life?
◯ Not at all ◯ A little ◯ Moderately ◯ Very much ◯ Extremely

n5. How much do you enjoy life?
◯ Not at all ◯ A little ◯ Moderately ◯ Very much ◯ Extremely

n6. To what extent do you feel that your life has meaning?
◯ Not at all ◯ A little ◯ Moderately ◯ Very much ◯ Extremely

n7. How well are you able to concentrate?
◯ Not at all ◯ A little ◯ Moderately ◯ Very much ◯ Extremely

n8. How safe do you feel in your daily life?
◯ Not at all ◯ A little ◯ Moderately ◯ Very much ◯ Extremely

n9. How healthy is your physical environment (climate, noise, pollution, attractions)?
◯ Not at all ◯ A little ◯ Moderately ◯ Very much ◯ Extremely

The following questions ask how fully you have felt or been able to do certain things over the last 2 weeks.

n10. Do you have enough energy for your day-to-day life?
◯ Not at all ◯ A little ◯ Moderately ◯ Very much ◯ Completely

n11. Are you able to accept your physical appearance?
◯ Not at all ◯ A little ◯ Moderately ◯ Very much ◯ Completely

n12. Do you have enough money to meet your needs?
◯ Not at all ◯ A little ◯ Moderately ◯ Very much ◯ Completely

n13. How readily available is the information you need in your day-to-day life?
◯ Not at all ◯ A little ◯ Moderately ◯ Very much ◯ Completely

n14. To what extent do you have opportunities for leisure activities?
◯ Not at all ◯ A little ◯ Moderately ◯ Very much ◯ Completely

The following questions ask how well or satisfied you have felt about various aspects of your life over the last 2 weeks.

n15. How well are you able to get around?
◯ Very poor ◯ Poor ◯ Neither poor nor good ◯ Good ◯ Very good

The following questions ask how satisfied you have felt about various aspects of your life over the last 2 weeks.

n16. How satisfied are you with your sleep?
- ◯ Very dissatisfied
- ◯ Dissatisfied
- ◯ Neither satisfied nor dissatisfied
- ◯ Satisfied
- ◯ Very satisfied

n17. How satisfied are you with your ability to carry out your day-to-day activities?
- ◯ Very dissatisfied
- ◯ Dissatisfied
- ◯ Neither satisfied nor dissatisfied
- ◯ Satisfied
- ◯ Very satisfied

n18. How satisfied are you with your ability to work?
- ◯ Very dissatisfied
- ◯ Dissatisfied
- ◯ Neither satisfied nor dissatisfied
- ◯ Satisfied
- ◯ Very satisfied

n19. How satisfied are you with yourself?
- ◯ Very dissatisfied
- ◯ Dissatisfied
- ◯ Neither satisfied nor dissatisfied
- ◯ Satisfied
- ◯ Very satisfied

n20. How satisfied are you with your personal relationships (friends, relatives, acquaintances, colleagues)?
- ◯ Very dissatisfied
- ◯ Dissatisfied
- ◯ Neither satisfied nor dissatisfied
- ◯ Satisfied
- ◯ Very satisfied

n21. How satisfied are you with your sex life?
- ◯ Very dissatisfied
- ◯ Dissatisfied
- ◯ Neither satisfied nor dissatisfied
- ◯ Satisfied
- ◯ Very satisfied

n22. How satisfied are you with the support you receive from your friends?

◯ Very dissatisfied
◯ Dissatisfied
◯ Neither satisfied nor dissatisfied
◯ Satisfied
◯ Very satisfied

n23. How satisfied are you with the conditions where you live?

◯ Very dissatisfied
◯ Dissatisfied
◯ Neither satisfied nor dissatisfied
◯ Satisfied
◯ Very satisfied

n24. How satisfied are you with your access to health services?

◯ Very dissatisfied
◯ Dissatisfied
◯ Neither satisfied nor dissatisfied
◯ Satisfied
◯ Very satisfied

n25. How satisfied are you with your means of transportation?

◯ Very dissatisfied
◯ Dissatisfied
◯ Neither satisfied nor dissatisfied
◯ Satisfied
◯ Very satisfied

The following questions refer to how often you have felt or experienced certain things over the last 2 weeks.

n26. How often do you have negative feelings, such as low mood, despair, anxiety, or depression?

◯ Never ◯ Sometimes ◯ Often ◯ Very frequent ◯ Always

n27. Did this questionnaire lead you to reflect on your role as a researcher or staff member at *IF Goiano*?

◯ Completely ◯ A great deal ◯ Partially ◯ A little ◯ Not at all

n28. Is there any final comment or any topic not addressed in this questionnaire that you would like to mention?

____

____

____

____

Thank you very much for your participation!
We will share the results of our study soon.
