# Supplementary material for: Researcher profiles, proposal submission, and project approval: an exploratory case study at a Brazilian Federal Institute
Source: Front Res Metr Anal. 2026 Oct 5;11:1888607. doi: 10.3389/frma.2026.1888607 (PMC13677698; doi:10.3389/frma.2026.1888607)
Supplement: Supplementary file 3 [file Table_1.pdf]

Supplementary Table 1. Complete distribution of respondent-reported proposal submission and approval of at least one project among submitters, according to researcher-profile characteristics.

Parameters	At least one proposal submitted		*p*-value	Effect size	At least one approved project among submitters		*p*-value	Effect size
	No	Yes			No	Yes		
Age group								
28–33 years	6 (37.5)	10 (62.5)	0.664^FH^		6 (60.0)#	4 (40.0)	**0.011**^FH^ *	0.403
34–39 years	6 (25.0)	18 (75.0)			1 (5.6)	17 (94.4)#		
40–45 years	5 (22.7)	17 (77.3)			7 (41.2)	10 (58.8)		
≥ 46 years	4 (19.0)	17 (81.0)			6 (35.3)	11 (64.7)		
Gender								
Female	11 (34.4)	21 (65.6)	0.132^P^		7 (33.3)	14 (66.7)	0.897^P^	
Male	10 (19.6)	41 (80.4)			13 (31.7)	28 (68.3)		
Ethnicity								
White	10 (22.7)	34 (77.3)	0.567^P^		9 (26.5)	25 (73.5)	0.283^P^	
Black/Mixed	11 (28.2)	28 (71.8)			11 (39.3)	17 (60.7)		
Marital status								
With a partner	14 (23.0)	47 (77.0)	0.412^P^		16 (34.0)	31 (66.0)	0.755^F^	
Without a partner	7 (31.8)	15 (68.2)			4 (26.7)	11 (73.3)		
Children								
No	9 (29.0)	22 (71.0)	0.546^P^		7 (31.8)	15 (68.2)	0.956^P^	
Yes	12 (23.1)	40 (76.9)			13 (32.5)	27 (67.5)		
Occupation at *IF Goiano*								
Teacher	18 (24.7)	55 (75.3)	0.708^F^		16 (29.1)	39 (70.9)	0.199^F^	
Administrative technician	3 (30.0)	7 (70.0)			4 (57.1)	3 (42.9)		
Employment relationship with *IF Goiano*								
External collaborator	–	3 (100.0)	0.568^F^		–	3 (100.0)	0.545^F^	
Public servant	21 (26.3)	59 (73.8)			20 (33.9)	39 (66.1)		
The weekly workload at *IF Goiano*								
≤ 40 hours	13 (33.3)	26 (66.7)	0.113^P^		7 (26.9)	19 (73.1)	0.445^P^	
> 40 hours	8 (18.2)	36 (81.8)			13 (36.1)	23 (63.9)		
Other roles at *IF Goiano*								
No	15 (31.9)	32 (68.1)	0.113^P^		10 (31.3)	22 (68.8)	0.861^P^	
Yes	6 (16.7)	30 (83.3)			10 (33.3)	20 (66.7)		
Work at other institutions								
No	20 (26.3)	56 (73.7)	0.673^F^		19 (33.9)	37 (66.1)	0.654^F^	
Yes	1 (14.3)	6 (85.7)			1 (16.7)	5 (83.3)		
Workload at other institutions								
≤ 20 hours	1 (20.0)	4 (80.0)	1.000^F^		–	4 (100.0)	0.333^F^	
> 20 hours	–	2 (100.0)			1 (50.0)	1 (50.0)		
Education level								
Master's degree	3 (42.9)	4 (57.1)	**0.020**^FH^ *	0.285	2 (50.0)	2 (50.0)	0.274^FH^	
Doctorate	17 (30.9)	38 (69.1)			14 (36.8)	24 (63.2)		
Doctorate with postdoctoral	1 (4.8)	20 (95.2)#			4 (20.0)	16 (80.0)		
Studies abroad								
No	18 (29.5)	43 (70.5)	0.142^P^		17 (39.5)	26 (60.5)	0.065^P^	
Yes	3 (13.6)	19 (86.4)			3 (15.8)	16 (84.2)		
Teaching in a postgraduate program								
No	18 (42.9)#	24 (57.1)	**< 0.001**^P^ *	0.409	10 (41.7)	14 (58.3)	0.208^P^	
Yes	3 (7.3)	38 (92.7)#			10 (26.3)	28 (73.7)		

Parameters	At least one proposal submitted		p-value	Effect size	At least one approved project among submitters		p-value	Effect size
	No	Yes			No	Yes		
Time as a researcher								
≤ 10 years	15 (28.3)	38 (71.7)	0.403[P]		15 (39.5)	23 (60.5)	0.126[P]	
> 10 years	6 (20.0)	24 (80.0)			5 (20.8)	19 (79.2)		
Participation in research projects								
No	2 (66.7)	1 (33.3)	0.156[F]		1 (100.0)	–	0.323[F]	
Yes	19 (23.8)	61 (76.3)			19 (31.1)	42 (68.9)		
CNPq PQ								
No	21 (28.4)	53 (71.6)	0.103[F]		20 (37.7)#	33 (62.3)	**0.047**[F] *	0.284
Yes	–	9 (100.0)			–	9 (100.0)#		
Weekly time allocated to teaching								
< 5 hours	–	–	0.317[FH]		–	–	0.237[FH]	
5–10 hours	2 (13.3)	13 (86.7)			4 (30.8)	9 (69.2)		
11–20 hours	9 (23.1)	30 (76.9)			7 (23.3)	23 (76.7)		
≥ 21 hours	10 (34.5)	19 (65.5)			9 (47.4)	10 (52.6)		
Weekly time allocated to research								
< 5 hours	3 (37.5)	5 (62.5)	0.329[FH]		2 (40.0)	3 (60.0)	0.250[FH]	
5–10 hours	10 (34.5)	19 (65.5)			9 (47.4)	10 (52.6)		
11–20 hours	4 (16.0)	21 (84.0)			6 (28.6)	15 (71.4)		
≥ 21 hours	4 (19.0)	17 (81.0)			3 (17.6)	14 (82.4)		
Weekly time allocated to extension programs								
< 5 hours	14 (30.4)	32 (69.6)	0.229[FH]		8 (25.0)	24 (75.0)	0.406[FH]	
5–10 hours	4 (14.3)	24 (85.7)			10 (41.7)	14 (58.3)		
11–20 hours	3 (33.3)	6 (66.7)			2 (33.3)	4 (66.7)		
≥ 21 hours	–	–			–	–		
Participation in international cooperation								
No	17 (30.4)	39 (69.6)	0.127[P]		16 (41.0)	23 (59.0)	0.054[P]	
Yes	4 (14.8)	23 (85.2)			4 (17.4)	19 (82.6)		
Publications in international cooperation								
No	1 (14.3)	6 (85.7)	1.000[F]		2 (33.3)	4 (66.7)	0.270[F]	
Yes	3 (15.0)	17 (85.0)			2 (11.8)	15 (88.2)		
International cooperation funding								
No	17 (30.4)	39 (69.6)	0.127[P]		16 (41.0)	23 (59.0)	0.054[P]	
Yes	4 (14.8)	23 (85.2)			4 (17.4)	19 (82.6)		
Projects submitted to foreign agencies								
No	21 (26.6)	58 (73.4)	0.568[F]		19 (32.8)	39 (67.2)	1.000[F]	
Yes	–	4 (100.0)			1 (25.0)	3 (75.0)		

Note: *n* refers to absolute frequency and % to relative frequency. For 'At least one proposal submitted', percentages were calculated using all respondents within each researcher-profile category. For 'At least one approved project among submitters', percentages were calculated among respondents who reported submitting at least one proposal during the preceding five years; the Yes column indicates those who reported at least one approved project and is not a proposal-level approval rate. *p*-values refer to Pearson's χ^2 tests when expected-frequency assumptions were adequate, two-sided Fisher's exact tests for sparse 2 × 2 tables, and Fisher–Freeman–Halton exact tests with Monte Carlo estimation when needed for larger sparse tables. Cramér's *V*, derived from the Pearson χ^2 statistic, is reported only for statistically significant associations as an unadjusted, descriptive measure of association. Cramér's *V* ranges from 0 (no association) to 1 (perfect association); conventional benchmarks were used: very small (< 0.10), small (0.10 to < 0.30), moderate (0.30 to < 0.50), and large (≥ 0.50); these classifications are approximate, and the estimates are descriptive (Cohen 2013); The symbol [#] identifies cells with positive adjusted standardized residuals ≥ 1.96, indicating observed frequencies higher than expected within statistically significant associations in post hoc residual analysis. [P] refers to Pearson's χ^2 test; [F] to Fisher's exact test; and [FH] to the Fisher–Freeman–Halton exact test. The *PQ*-holder category comprised 9 respondents in the full sample; therefore, interpret findings regarding PQ status with caution. Bold values marked with * indicate a statistically significant association ($\alpha = 0.05$).
